# Recent Advances in the Development of Biomimetic Materials

**DOI:** 10.3390/gels9100833

**Published:** 2023-10-20

**Authors:** Maria G. Ciulla, Alessio Massironi, Michela Sugni, Matthew A. Ensign, Stefania Marzorati, Mahdi Forouharshad

**Affiliations:** 1 Department of Chemistry, Università degli Studi di Milano, Via C. Golgi 19, 20133 Milan, Italy; 2 Department of Environmental Science and Policy, Università degli Studi di Milano, Via Celoria 2, 20133 Milan, Italy; 3 Fischell Department of Bioengineering, University of Maryland, College Park, MD 20742, USA; 4 Department of Ophthalmology, Wilmer Eye Institute, Johns Hopkins University School of Medicine, Baltimore, MD 21287, USA

**Keywords:** biomimetics, biocompatibility, tissue engineering, mechanical properties, biomimetic hydrogels, collagen, self-assembling peptides, surface modification, extracellular matrix, 3D bioprinting

## Abstract

In this review, we focused on recent efforts in the design and development of materials with biomimetic properties. Innovative methods promise to emulate cell microenvironments and tissue functions, but many aspects regarding cellular communication, motility, and responsiveness remain to be explained. We photographed the state-of-the-art advancements in biomimetics, and discussed the complexity of a “bottom-up” artificial construction of living systems, with particular highlights on hydrogels, collagen-based composites, surface modifications, and three-dimensional (3D) bioprinting applications. Fast-paced 3D printing and artificial intelligence, nevertheless, collide with reality: How difficult can it be to build reproducible biomimetic materials at a real scale in line with the complexity of living systems? Nowadays, science is in urgent need of bioengineering technologies for the practical use of bioinspired and biomimetics for medicine and clinics.

## 1. Introduction

Can science imitate life? Nature has always inspired researchers to assess principles for the design of sophisticated models and architectures of human life [1]. During 3.8 billion years, evolution has refined living organisms, systems, materials, and processes to create the most efficient arrays of products [2]. Therefore, biomimicry can take lessons regarding the secret laws that govern the perfect machines of our biological systems. Biomimetic materials are developed, thus, to emulate and replicate one or more attributes of a living organism, to restore a natural function, or to sustain an environment in terms of chemistry, processing, and structure of materials [3]. Biomimetic science is not restricted to biomaterials engineering but instead, is referred to a wide variety of applications. Functional biomimicry, process biomimicry, molecular biomimicry, and structure biomimicry identify the context of the benefit. Restoring and maintaining the normal functions of a damaged tissue or organ is the aim of modern tissue engineering. Nevertheless, due to its complexity, this field involves the knowledge of multidisciplinary areas, including chemistry, physics, materials science, biology, medicine, bioengineering, and biotechnology [4]. An enormous clinical need exists for the development of biomaterials to provide structural support in terms of mechanical properties and tissue growth (i.e., cell attachment) and proliferation, to present biologically active signals (e.g., growth factors), and to allow in vivo cell migration in a transplanted organ. The key element of the rationale for building an efficient and usable biomimetic material lies in the emulation of the extracellular matrix (ECM) and the related local microenvironment. Due to this, ideal materials for biological use resembling ECM microenvironments are continuously studied. Despite significant progress in bioengineering, clinical translation faced limitations due to the inevitable variability in patients. To achieve the required goals, scientists must consider minimizing the invasive techniques, as well as involve the use of cell populations and bioactive factors and to develop tailor-made and patient-specific biomaterials [5]. The important role of mechanical properties, as a key point for the design of tissue-engineering constructs, demands strengthened attention, and very little success has been achieved [6]. Hydrogels, 3D-polymeric substances, can act as a scaffold and can the mimic properties of ECM and tissues [7]. Their programmable reactivity to specific stimuli (pH, temperature, ionic strength, electromagnetic field, and light) make hydrogels suitable for the development of biomimetics. In the class of hydrogels, self-assembling peptides (SAPs) hold a large space in tissue engineering and biomedical applications [8].

During the decades, a wide number of materials were discovered and studied to be used as biomimetics. Surface interactions at the biomaterial–cell interface play crucial roles in cell behaviors, such as adhesion, proliferation, and differentiation. Some properties of biomaterials, including topography (roughness and pores), physical (mechanical properties and wettability), and chemical (surface energy and charge, bioactive molecule) have been shown to have a significant effect on biomaterial–cell interaction.

In this wide background, the present review is organized as a comprehensive discussion regarding the most recent advancement as well as the most promising trends in terms of materials and techniques. 

**Scheme 1:**
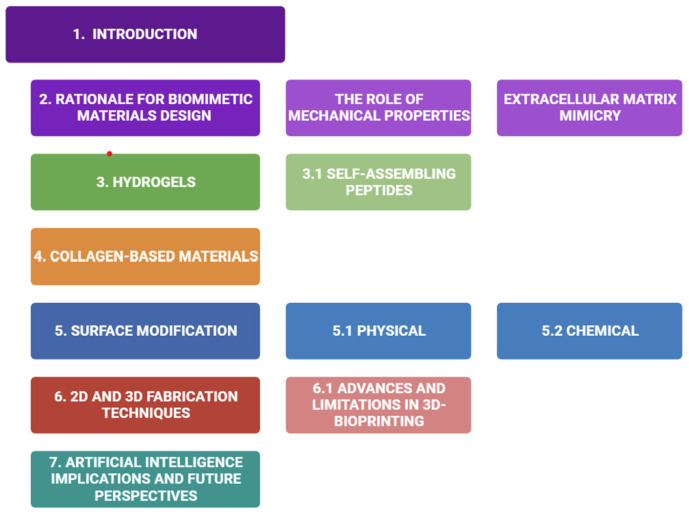
Representative diagram of the content of this review.

Scheme 1 displays a representative diagram of the content of this review. In detail, the first section is dedicated to the rationale for biometic materials design, how to overcome the difficulties of efficient biocompatibility, following the need for proper mechanical properties while mimicking the extracellular matrix components and functions. Then, a specific focus will be set on hydrogels and their different fabrication techniques, depending on chemistry beyond the choice of their constituents’ materials, including self-assembly peptides. A section is dedicated to collagen-based biomaterials and their properties depending on the bio-based source for their extraction, impacting on the final morphological and biological features. 

Another section follows with physical and chemical surface modifications, as the primary interaction between the biomaterial and the living tissues is represented by the surface itself and hence, is responsible for the primary communication behavior. The last sections are then dedicated to the most recent advancements in 3D bioprinting, capable of creating biological structures by layering biomaterials in a precise and controlled manner, and artificial intelligence, a promising tool able to assist and rationalize biomaterials development.

## 2. Rationale for Biomimetic Materials Design 

### 2.1. The Role of Mechanical Properties 

The studies on biological systems considered as engineering structures date back to the 1970s with works from D.W. Thompson [9] and J.D. Currey [10]. In many biological systems, it is possible to find mechanical properties that are hard to identically reproduce in synthetic materials [11]. This is quite curious considering that biological architectures are made of polymers with simple elements, such as C, N, H, and O.

**Figure 1:**
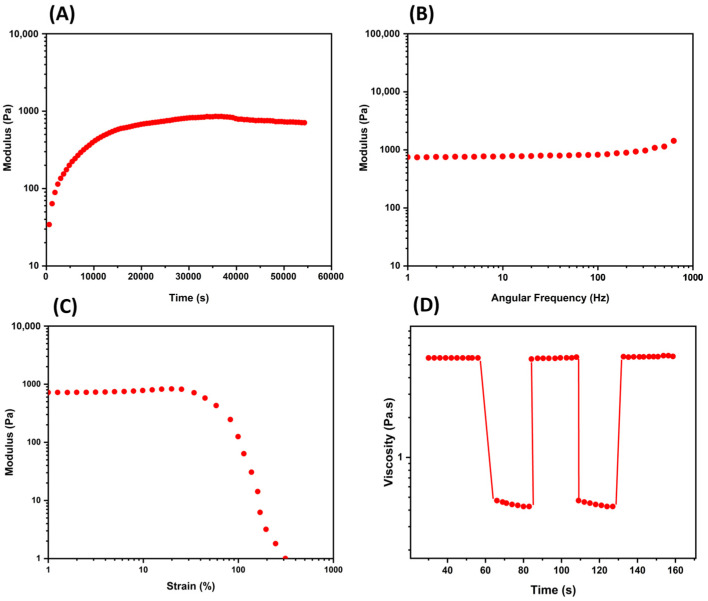
Mechanical property characterization using common rheological experiments. (**A**) The time sweep test (oscillatory) provides information regarding the change in a hydrogel over the time at constant oscillation; (**B**) the frequency sweep test (oscillatory) relates the frequency and the storage and loss moduli in a range of frequency; (**C**) the strain sweep test is conducted in an increasing oscillatory strain at constant frequency to examine the linear viscoelastic region (Newtonian behavior); (**D**) a thixotropy graph is used to study non-Newtonian behavior with continuous oscillatory strains and indicating the self-healing of a hydrogel. Other rheological evaluations are given by temperature ramps, creep and recovery tests, and hysteresis loops.

Mechanical properties of the tissues, as of all other materials, are characterized by the relationship between the force applied to the material and the related extension, measured in terms of resistance to deformation and shape changes. The elastic modulus (*E*, or Young’s modulus) represents the stiffness of a material, and it is the slope of the stress–strain curve [12]. A stiffer material (higher elastic modulus, i.e., bones) is less deformable than one characterized by a lower elastic modulus (i.e., soft biological tissues, such as skin, cartilage, and heart valves) [13]. Table 1 lists measured elastic moduli of tissues and materials, which can be obtained mainly using atomic force microscopy (AFM) and rheological experiments (Figure 1) [14].

Above all, human tissues are elastic: this irreplaceable property is given by elastin [35], an ECM protein that confers not only extendibility and resiliency but also some sort of resistance [36]. Human native elastin biosynthesis takes place during the 17th week of pregnancy and continues into the early neonatal stage [37]. During its long half-life (about 70 years), elastin can be extended and relaxed billions of times without losing its functions [38]. After an injury, elastin is produced under the influence of exogen factors (i.e., tumor necrosis factor-a, interleukin 1b, insulin-like growth factor 1, and transforming growth factor), but the resulting synthesized elastin is not the same as the native as it appears more disorganized [37].

The never-ending quest for the elastomeric property is pushing researchers to confer this physical performance to polypeptide sequences in order to emulate living systems, and a vast number of studies in the literature agree on the potential use of elastin-like peptides (ELPs) for biomedical applications [21,39,40,41].

An important property of elastin fibers is extendibility, which consists of the ability to become more than twice as long before rupture and to return to the original dimensions when tension is released. On the other hand, elastic hysteresis (repetitive cyclic loading) occurs with a viscoelastic material. A more resilient elastomeric tissue will exhibit a lower hysteresis [12]. Collagen, the structural protein found in connective tissue, is the second element of elastic tissues. While elastin provides elasticity, collagen fibers confer strength and structural support. Its biological function is related to its mechanical properties [34]. Despite collagen being extensively characterized, a question regarding the comprehension of structural models for fibrils and fibers remains open.

Peptide-based materials were designed to mimic the properties of elastin and collagen, so they can be used in regenerative medicine and tissue engineering applications [21,42]. As they are formed of amino acid residues, they possess the considerable advantages of low immunogenicity and biodegradability in non-toxic metabolites [43].

ELPs are polymers made on the sequence of tropoelastin, the precursor of elastin, which contains alternating hydrophobic and hydrophilic residues [21]. ELP sequences are usually composed of hydrophobic repeats of Val-Pro-Gly-X-Gly (where X is any amino acid except Pro). They are faced with thermal responsivity, resulting in being soluble below a characteristic transition temperature [44]. An interesting point is that ELPs can be fine-tuned on the basis of requested physical properties, chemical reactivity, self-assembly behavior, and biological activity; thus, they can be conjugated with drugs, ligands, imaging agents, and crosslinkers. It was reported that ELPs support the formation of ECM in articular cartilage by other cell types and in different cell culture conditions, such as with chondrocyte differentiation of human adipose-derived adult stem (hADAS) cells [45]. Due to the poor mechanical properties of non-crosslinked ELPs compared with cartilage, which make ELPs unsuitable for implantation in a cartilage defect, crosslinked ELPs were designed to provide stiffer materials. Trabbic-Carlson et al. produced a genetically engineered ELP with Val-Pro-Gly-X-Gly repeats, where X was Val or Lys (every 7 or 17 pentapeptides) [46]. Then, they chemically crosslinked this ELP with tris-succinimidyl aminotriacetate, affording a hydrogel with a stiffness of 1.6 to 15 kPa at 37 °C (based on ELP concentration and Lys content). In 2007, another work demonstrated that Lys-rich ELPs can quickly form hydrogel thanks to the crosslinking with β-[tris(hydroxymethyl)phosphino]propionic acid (THPP) under physiological conditions [47]. In rabbits, genipin was crosslinked with an ELP before implantation to provide a stiff gel with similar aggregation to that of cartilage [48]. In 2018, Mozhdehi and co-workers developed an ELP functionalized with a C_14_ fatty-acid molecule in a post-translational modification [49]. The resulting hybrid biomaterial exhibited temperature-triggered hierarchical self-assembly with tuning properties typical of amphiphilic peptides. Further and extensive ELP modifications are described by Varanko et al. in an excellent review [21]. 

Improvements in the stiffness of materials for biomedical applications can be achieved with the use of chemical or physical crosslinkers [25,30,50,51,52,53,54,55] as well as enzymatic techniques [56]. Nevertheless, their application is sometimes limited due to the toxicity of the crosslinker. Drawing inspiration from natural bone and tissues, in 2022, Gelain and co-workers developed a double crosslinking based on 4-(N-Maleimidomethyl) cyclohexane-1-carboxylic acid 3-sulpho-N-hydroxysuccinimide ester (Sulfo-SMCC) and genipin with cysteine and lysine-rich LKLK12 SAPs [25]. The resulting hydrogels reached values higher than 800 kPa, an impressive achievement in the field of SAPs. Recently, Ciulla et al. reported a sustainable and cell-compatible crosslinking of SAPs with the use of low-power microwave (MW) irradiation, reaching modest to high values of stiffness and viscoelasticity [57]. Despite some steps forward that have been made, significant efforts are yet necessary to reach stiff and viscoelastic biomaterials.

### 2.2. Extracellular Matrix Mimicry 

An artificial biomimetic scaffold for tissue engineering should provide properties of the natural ECM in order to allow cell regeneration and support cell adhesion, proliferation, differentiation, and neo-tissue formation [58,59,60,61]. Cell adhesion is a prerequisite for the regulation of fundamental cellular processes. Hence, it is a crucial task to control the adhesion of cells on biomaterials, which can be achieved with surface modification as the only intimate contact with the biological environment. As a dynamic component of all tissues, the desired ECM-like scaffold architecture should have a 3D geometry with a specific porosity necessary for migration and cell proliferation, as well as for nutrition and degradation product transition. The ability of a tissue’s mechanical strength depends, above all, on the matrix composition related to tissue function [12]. Vascularization and perfusion are other fundamental prerequisites for cell engraftment. In this regard, endothelial and vascular endothelial growth factor (VEGF) may be added to the new scaffold. 

On the other hand, the assembly of tailored fibrous structural proteins is wired by the polymerization of ECM monomers into fibers (fibrils in the case of collagen) [62]. Fibrillar collagens assemble from triple helices of tropocollagen in fibrils (10–300 nm diameter) and then in fibers (1–20 µm). The tensile strength of tissues depends on a peculiar arrangement of fibrils, formed by different types of collagens in a single fibril [62]. In tendons, for example, fibers assemble into bundles (~500 µm). The interaction between fibrils and microenvironments is dominated by the dense heterogeneity in other specific macromolecules, including proteoglycans (i.e., hyaluronic acid and glycosaminoglycans), amino acids (mainly Pro and Gly), and elastin. As described by the Hodge and Petruska model [63], individual collagen monomers are aligned in a fibril, followed by a pseudo-periodicity made of a characteristic overlap gap. Collagen distribution provides strength, yielding bio-mechanical requirements typical of a tissue [64]: while collagen type I confers stiffness, collagen type III is more flexible. In summary, extracellular networks dictate the rules for the rational design of a resilient biomimetic material. ECM composition and assembly allow tissues to comply with different and sophisticated biological functions. Abune et al. recently reported the development of a dual aptamer-functionalized hydrogel able to bind cells and sequester growth factors with the use of a vascular endothelial growth factor binding aptamer (VEGF aptamer) and a c-MET receptor binding aptamer (c-MET aptamer), providing a synergistic effect on cell survival and proliferation [65]. 

In bioartificial biomimetic materials, several strategies can be adopted to build a scaffold able to direct cell responses, including the incorporation of cell binding motifs (e.g., RGD motif), the functionalization with polysaccharides (to enhance cell-material interactions), and the incorporation of decellularized ECM into the scaffold (to mimic a very close microenvironment to natural niches).

## 3. Hydrogels 

Hydrogels are porous 3D networks constituted by hydrophilic synthetic or natural polymers, with the ability to absorb and retain water or biological fluids while maintaining a distinct 3D structure. The amount of solvent absorbed into the hydrogel is dependent on the temperature, pH, and a specific interaction between the functional groups of polymer chains and water molecules. Their stability is provided by the presence of chemical or physical crosslinks between the polymeric chains, which allow the 3D network to swell and not dissolve in an aqueous environment [66]. Thanks to their unique nature, hydrogels find several applications spanning industrial and biomedical fields, such as drug delivery systems [66], pH sensors [67], scaffolds for tissue engineering [68], contact lens preparation [69], and biosensor and supercapacitor fabrication [70]. Among different investigated areas, the biomedical field represents the most exploited application of hydrogels due to the high affinity to water that allows for maintaining all the metabolic activities of the organisms. Hydrogels can be designed for selective permeability, mechanical and chemical stability, and a lifetime inside the human body [71], making them a unique tool, especially in regenerative medicine, wound dressings, drug delivery, and hygiene products [72,73]. Moreover, hydrogels can mimic ECM composition and/or organization to achieve biological and mechanical properties similar to those of native tissues using non-invasive surgical procedures; since the tissue area is usually irregular, incomplete area coverage would lead to delayed healing and bacterial infections [74]. Therefore, hydrogels can ensure complete cover over the wound bed through rapid shape adaptability. The strong affinity of hydrogels for aqueous environments makes them ideal for developing 3D structures. Biomimetic hydrogels are engineered to replicate specific characteristics found in living organisms, and such features can vary depending on the intended application and target tissue [75].

As described in Table 2, the classification of hydrogels is based on their source (natural or synthetic), charge (non-ionic, ionic, zwitterionic), polymeric composition, physical properties, and the crosslinking mechanism.

Among different materials, natural-based hydrogels have gained significant interest due to their inherent biocompatibility and cost advantages over synthetic materials. Proteins, polysaccharides, and peptide-based hydrogels are mainly known for their biocompatibility with host organisms during in vivo experiments, but they have the ability to easily simulate the ECM. Protein-based hydrogels, like collagen and silk fibroin, possess functional groups suitable for forming hydrogels, providing an advantage over polysaccharides that often require chemical modifications for stable crosslinking [61]. Polysaccharide-based hydrogels, such as glycosaminoglycans and chitosan, exhibit excellent biological properties due to their structural similarities with biological molecules [77]. Among polysaccharides, cellulose represents a unique and promising biomaterial for hydrogel formulation. Cellulose can derive from sources like wood pulp and cotton and also microorganisms such as *Acetobacter xylinum*, gaining significant attention due to its unique properties provided by the combination of amorphous and crystalline regions [94,95]. This rod-shaped aerobic bacterium was found to be responsible for the production of high-yield cellulose as microfibrils on a large scale and it is considered to be a model organism for the production of microbial cellulose for commercial fermentation. Apart from this group, cellulose can be synthesized by other bacterial genera such as *Agrobacterium* spp., *Acetobacter* spp., *Azotobacter*, *Rhizobium* spp., *Sarcina*, *Alcaligenes*, and *Pseudomonas* [96]. Cellulose can be modified to create cellulose-based hydrogels with tuneable properties, such as porosity, mechanical strength, biocompatibility, and interaction with hydrophilic or hydrophobic biopolymers while being used in the biomedical domain. In this context, cellulose offers a greener alternative to traditional synthetic polymers and other polysaccharide-based materials, which commonly lack hydrophobicity degree and mechanical strength [94]. With its versatility, abundance, and eco-conscious profile, plant-based and microbial-origin cellulose represents an essential biomaterial for hydrogel preparation [97]. Moreover, bacterial cellulose can be found in micro/nanocrystalline forms with purity not requiring further processing to remove other molecules such as lignin and hemicellulose. Micro/nanostructured celluloses have recently been shown to significantly enhance the stability and mechanical performance of hydrogels and endow them with interesting biological features [96]. Their hydrophilicity is provided by the presence of several hydroxyl groups on its surface, allowing easy chemical functionalization and providing most of its biological features.

Biomimetic hydrogels must possess fundamental features to simulate the ECM including: wettability—exhibiting a high water content similar to many biological tissues and fluids enhances their biocompatibility and affinity for biological systems; biocompatibility—hydrogels must not exhibit cytotoxicity once inserted in human body, making them suitable for in vivo applications; swelling behavior—replicating the swelling and deswelling of natural tissues, enabling controlled drug release and wound healing activities; mechanical properties—matching the properties of specific tissues, including elasticity and stiffness; porosity and permeability—controlled pore structures in biomimetic hydrogels mimic the ECM, supporting nutrient diffusion; molecular recognition—the ability to enable selective binding to specific molecules such as growth factors promoting cell adhesion, proliferation, and differentiation, facilitating tissue engineering; and stimuli responsiveness—hydrogels can be designed to respond to pH or temperature changes, mirroring natural pH and/or temperature regulation in specific microenvironments [98]. For these reasons, in order to replicate ECM physicochemical properties, natural-based hydrogels are commonly tuned and modified to emulate native cellular microenvironments. 

The Inherent challenge lies in controlling the chemical and physical properties of ECM natural proteins. Conversely, synthetic materials like poly(lactic-co-glycolic acid), poly(urethane), and biodegradable glass offer enhanced control over these properties, but they often exhibit reduced compatibility with essential cellular functions such as growth, differentiation, and tissue formation [68].

HA-based hydrogels have gained widespread utilization of ECM scaffolds, aimed at enhancing or restoring biological functions. The HA’s natural presence in the ECM boasts high biocompatibility and serves vital roles in cellular signaling like cell adhesion, migration, and proliferation, primarily mediated through interactions with extracellular molecules such as CD44 and RHAMM [99]. Furthermore, HA offers cells a favorable 3D microenvironment, closely mirroring the native ECM in hydrogel form. This microenvironment can be readily tailored to suit specific tissues using chemical modifications and the inclusion of various biomaterials, stem cells, and fabrication techniques [100]. Consequently, HA-based hydrogels exhibit substantial promise in the realm of tissue engineering and regeneration of tissues abundant in HA, including cartilage, bone, skin, and brain tissue [100]. However, weak mechanical properties combined with rapid degradation and clearance in vivo limit the medical applications of pristine HA [101]. In this respect, to improve HA mechanical properties and reduce its fast biodegradation, the polysaccharide structure has been modified by introducing new functional groups such as aldehyde, hydrazide, thiol, furan, and methacrylate, which allow for forming stable HA hydrogels through covalent crosslinking [102], enhancing it biomimetic activities.

The evolution in biomaterial design aims to combine the advantageous attributes of both natural and synthetic systems to create materials that facilitate crucial cellular processes, including attachment, migration, proliferation, and differentiation, while also enabling the efficient diffusion of waste and nutrients within the scaffolds. In pursuit of this goal, researchers have developed various materials, including those founded on self-assembling peptides [103]. Peptide hydrogels are a sub-group of natural hydrogels that are extremely attractive for biomedical applications [104]. The cost of peptide synthesis is comparable to some polymeric hydrogels, but in terms of tuning peptide synthesis, reaction conditions, and purification steps, peptide hydrogels are superior to other synthetic-based hydrogels [104]. This class of hydrogels is commonly obtained using solid-phase synthesis, allowing the development of secondary structures such as α-helix and β-sheet hydrogels. Moreover, there is increasing evidence that the incorporation of peptides within regenerative hydrogels can result in the generation of structural recognition motifs that can enhance cell attachment or induce cell signaling pathways, improving cell infiltration or promoting a variety of other modulatory biochemical responses [105]. 

### Self-Assembling Peptides

**Figure 2:**
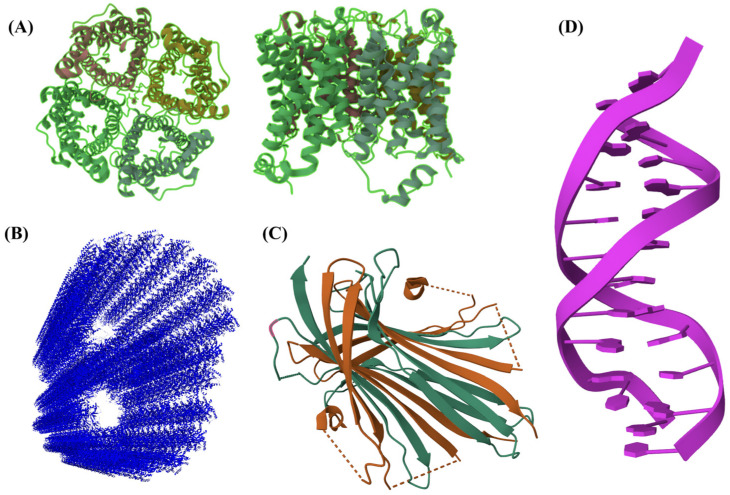
Supramolecular architectures. (**A**) Human aquaporin 5 X-ray crystal structure (PDB ID: 3D9S) having a tubular conformation, acting as a channel for the transport of water across cell membranes [133]. (**B**) Ciliary doublet microtubule (electron microscopy 3.4 Å, PDB ID: 6U42): protofilaments of microtubule assemble playing fundamental roles in eukaryotic cells [134,135]. (**C**) X-ray diffraction of the N-terminal domain of Bombyx mori fibroin (PDB ID: 3UA0) that mediates silk assembly with a remarkable antiparallel β-sheet content [136]. (**D**) The assembly of a double helix of DNA through the pairing bases of complementary sequences (X-ray diffraction, PDB ID: 111D).

Self-assembly is a ubiquitous phenomenon that occurs spontaneously in nature and biology. Thanks to non-covalent and supramolecular interactions, two or more molecules can self-organize in well-ordered and complex architectures (Figure 2). These nanostructures include fibers, micelles, vesicles, nanotubes, and nanorods [106]. The ability to self-assemble makes SAPs ideal for biomedical applications: in fact, they possess all the advantages of peptides (non-toxicity, non-immunogenicity, and biodegradability) [43]. Moreover, the sol–gel transition provides a platform for the design of fine-tuning biomaterials, especially for wound healing and tissue engineering [107]. The first SAP, Ac-(AEAEAKAK)_2_-CONH_2_, was discovered in the yeast of protein zuotin, in 1993, by Shuguang Zhang [108]. Subsequently, other SAPs were developed: RADA16 [109,110], KLDL12 [111], biotin-based SAPs [112], Fmoc-FFF [53], LKLK12 [25,30], *all-L* cyclo-LDLK12 and *all-L* cyclo-FAQLDLK12 [113], *D/L* cyclo-FF [114], *D/L* cyclo-LDLD2 [115], branched-SAPs [51], amphiphilic SAPs [116,117], and oligoSAPs (O_n_(SL)_6_O_n_) [118].

As synthetic bioabsorbable materials, SAPs gained enormous interest in the last two decades [110]. Marchini et al. developed a 3D cell culture model of densely seeded human neural stem cells (hNSCs) using multifunctionalized hydrogels with neuroregenerative potential in vivo for the treatment of spinal cord injury [119]. 

Recently, by mimicking pancreatic islets seeding in ECM, linear and branched LDLK12-based SAPs were used for 3D-seeded human pancreatic islets (hPIs) for subsequent in vivo transplantation in nude diabetic mice [120]. 

Gelain and co-workers reported an in vivo study of biomimetic electrospun SAP micro-channels suitable to support NSC transplant in spinal cord injury [121]. Important results in the field of SAPs as biomaterials are emerging for cancer therapy [122,123], as immunomodulatory agents [124], and against antibiotic resistance [125], as well as for tissue regeneration, drug delivery [126], sensing [127,128], 3D printing [129], and nanomedicine [130,131,132].

Since 2014, self-assembly has been gaining attention, also in robotics [137], where researchers take inspiration from collective intelligence found in nature to create artificial abilities like in flocks of birds or in bees and ants. Similarly, robots with abilities to work in groups and cross enormous difficulties were realized, as well as self-assembling “cellular” robots that can act as a wrench in a programmable matter [138,139].

## 4. Collagen-Based Materials

Collagen is the most abundant fibrous protein in the mammalian body and, together with elastin and hyaluronic acid, constitutes the main component of the extracellular matrix (ECM). It is primarily responsible for the skin’s mechanical properties (tensile strength, elasticity, etc.) and overall integrity in most soft and hard tissues in animals [140,141]. It is organized in a highly hierarchical structure, the fundamental unit being a triple helix of polypeptide chains (tropocollagen). Numerous tropocollagen units can then be assembled in banded fibrils, and these last in larger supramolecular structures, the fibers [142]. In mammals, up to 29 types of collagens were identified, differing in polypetide chains and functionality (e.g., fibrils or network-forming, transmembrane, etc.). Among the different ones, type I collagen is the most abundant (80% of the skin collagen) and for this reason, it is often used in biomaterials fabrication [143]. For all these characteristics, when designing biomimicking scaffolds for tissue (particularly skin) regeneration, collagen is among the most popular biomaterials due to its excellent biocompatibility, wide distribution in tissues, and ability to mimic the natural ECM to facilitate cellular attachment, migration, and proliferation.

Regarding its industrial exploitation, as demonstrated by the past literature in the field of biomaterials engineering for skin regeneration applications, collagen is mainly of mammalian origins and generally isolated from the Achilles tendon, bones, or skin. 

Regarding mammalian collagen, Ghodbane and co-workers demonstrated that bovine, porcine, and ovine tendon-derived collagen could be easily collected at slaughterhouses without interfering with the meat harvesting process, with positive implications for the sustainability of the collection process. After a non-enzymatic collagen-extraction process yielding from 74 to 80% of the starting biomass, they produced scaffolds with similar overall physical and mechanical properties. The freeze-drying process, with fine control of the formation and sublimation of ice crystals, resulted in the formation of a highly porous sponge-like scaffold, sufficiently permeable with interconnecting pores to facilitate cell growth, migration, and nutrition. A crosslinking with EDC/NHS was used for further biomaterial stabilization [144]. 

Recently, Nayak et al. used emerging 3D printing technology to demonstrate the effectiveness of the printability of 3D-printed scaffolds with engineered pore architectures. Starting from a commercial bovine dermal collagen, the authors formulated a stable colloidal gel extruded using a 3D printer, followed by crosslinking. Scaffolds were stable and characterized by adequate mechanical properties, fundamental to producing self-standing materials upon extrusion. The 3D-printed scaffolds promoted cellular attachment and proliferation, making them a promising material for customized regenerative applications [145]. However, among the main concerns of animal-derived collagens, the first one is directly connected to religious and cultural constraints and, even more importantly, the second one is connected to the risks of infectious disease transmission, as observed in the case of bovine spongiform encephalopathy transmission via contaminated scaffolds [146]. In addition, clinical observations of allergies and immunogenicity have been reported, indicating that 2–4% of the population is allergic to porcine- and bovine-derived collagen [147].

In response to these sanitary concerns, other collagen sources are under consideration, and the literature of the last few years is increasingly investigating marine collagen, becoming a popular subject. Prioritizing sustainability and responsible sourcing practices, marine collagens can be obtained from different sources. Marine collagen sources include vertebrates (fish and marine mammals) and invertebrates (cuttlefish, squids, sea cucumbers, sea urchins, etc.). Regarding the first category, considering that up to 75% of fish weight is discarded during the industrial processing chain (skins, bones, fins, heads, guts, scales, etc.), researchers have reported the possibility of obtaining collagen from these wastes, with an important increase in the economic value of the by-products, thus offering both economic and environmental benefits [147]. 

It was reported that marine collagen differs from mammalian collagen in the levels of specific amino acids, with a significant variation among fish species, the temperatures at which the fish live, and thus, the thermal stability of the final extracted collagen [145]. This seems related to a different content of hydroxyproline residues, responsible of interchain hydrogen bond formation and thus contributing to the stabilization of the collagen triple-helix. As a consequence, marine collagen is responsible for some practical disadvantages compared with its mammalian counterpart, such as higher rates of degradation, poorer mechanical properties, and lower denaturation temperature [148]. To overcome these drawbacks and improve the performance of collagen-based hydrogels, different strategies are applied, many of them already known from the previous mammalian collagen literature, already attempting methods for collagen stabilization. Physical crosslinking is the first option, referring to physical effects ending in a crosslinked network. UV light irradiation is the most commonly used strategy, while temperature fine control (such as heating under vacuum or freeze-drying), removing absorbed water, is another option to form 3D network structures. Excessively high temperatures and excessively long exposure times might cause collagen degradation and scaffold collapse. For this reason, a photosynthetizer (Irgacure 2959) is often used to facilitate crosslinking and keep physical conditions milder to preserve biomaterial properties. The use of riboflavin, genipin, or other additives to improve crosslinking or the chemical modification to obtain crosslinked methacrylated collagen has also been reported, specifically when the production of a printable ink was the main goal [93,149,150]. 

**Figure 3:**
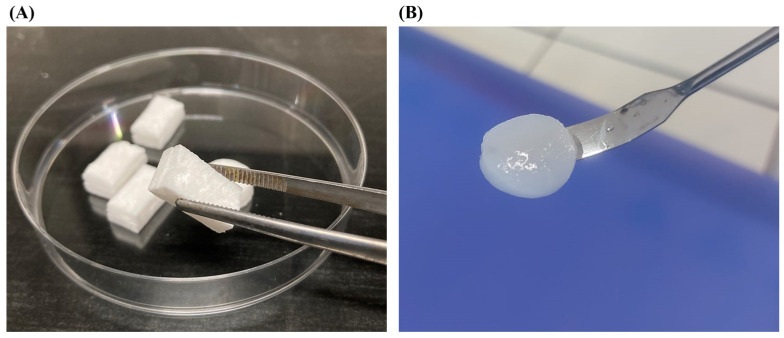
Sea urchin waste collagen biomaterials; courtesy of Sugni and Marzorati. (**A**) Lyophilized collagen-based scaffolds. (**B**) Collagen-based hydrogels.

One of the most impacting factors on the biological, chemical, and mechanical properties of the final scaffold is also related to the adopted extraction procedure of collagen from the original matrix. Enzymatic or acidic treatments are often used, resulting in the partial loss of the native fibrillar organization of collagen, collected in its hydrolyzed form. Even if collagen gelatin can be more easily manipulated for scaffold production purposes, the complete and perfectly functional fibril reconstruction can be difficult to achieve, ending up with even weaker mechanical properties and sub-optimal features [151,152]. Strictly connected to this, most of the procedures adopted in the literature fail in preserving the association of important components reproducing characteristics and functionality (for example, hydration provided by the presence of glycosaminoglycans) of the ECM and need to be artificially added post collagen extraction. This is the case of the “gold standard” Integra^®^, a commercially available product for skin wound healing, in which a bovine-derived scaffold is artificially enriched with shark-derived chondroitin sulfate (the most common GAG-like component of the dermis). In response to this, Ferrario and co-workers demonstrated the possibility of recovering collagen from sea urchin waste in its native fibrillar form, decorated with surface GAGs periodically organized along the fibril surface according to standard D-patterning [151,152]. It was demonstrated that mutable collagenous tissue (MCT) derived from echinoderms possesses biological peculiarities that facilitate native collagen extraction and use for biomedical applications such as regenerative purposes for the treatment of skin wounds [153]. In Figure 3, some pictures of marine collagen-based scaffolds are displayed.

Given the impossibility of maintaining the presence of GAGs in each extraction procedure, co-blending modification is an alternative strategy used to compensate for the deficiencies of collagen material by drawing on the advantages of other materials. Biocomposites prepared from collagen and HA can offer inherent advantages in mimicking the structure and functionalities of the natural extracellular matrix. The formation of proteoglycans mimicking materials, in which proteins are attached to GAG-like structures such as HA, conveys unique hydrating and mechanical properties. The presence of HA in scaffolds is demonstrated to enhance resistance to compressive forces, and HA cell receptors regulate functions such as chondrogenesis, osteogenesis, neurogenesis, cardiogenesis, and angiogenesis [154].

Despite the advantages of good biocompatibility and hydrophilicity, tissue engineering applications of collagen are restricted by weak mechanical properties and rapid degradation. Both these aspects are aligned with less strict requirements if the application is related to skin regeneration applications, in which scaffolds need to be soft and easily degradable to accompany the wound healing process. 

**Figure 4:**
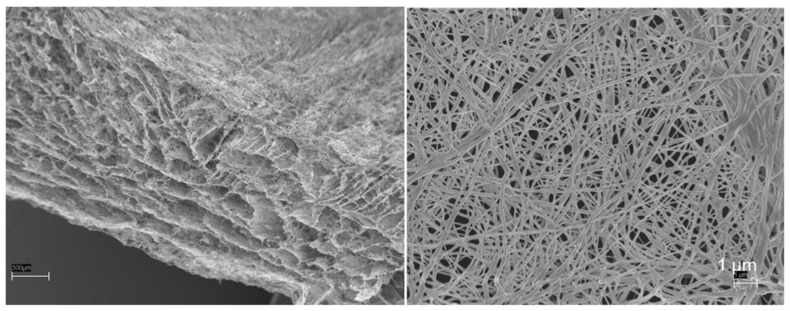
Sea urchin collagen scaffolds observed using SEM imaging at different magnification levels (courtesy of Sugni and Marzorati).

Figure 4 displays the SEM images of marine collagen-based lyophilized scaffolds, characterized by a highly porous structure, heterogeneous porous shape, and size throughout the full thickness with both lamellar and network structures. The resulting mean porosity appears suitable to allow cell seeding and infiltration. 

However, if the final application is connected to harder tissue regeneration, for example, bone regeneration, other strategies must be used because collagen itself is not able to provide the necessary mechanical properties. Since collagen and apatite minerals are the main components of bones, tissue engineering research has been investigating the possibility of combining these materials by using different biomimetic approaches [155].

The nature-inspired biomineralization process, in which organisms operate complex cascades of phenomena generating organic and inorganic hybrid nanostructures (such as bones whose main components are collagen and hydroxyapatite), is hence reproduced artificially in the lab [156]. In brief, in one of the most commonly adopted methods to produce bone-mimicking materials, collagen acidic solution containing phosphate ions is added dropwise and mixed with an alkaline solution containing calcium ions, hence promoting the nucleation, precipitation, and growth of nano-hydroxyapatite crystals within the collagen fibers [157]. In a work by Lickorish et al., the authors developed a collagen-hydroxyapatite composite as a bone graft substitute. The presence of hydroxyapatite was confirmed with X-ray diffraction, and in vitro cytotoxicity assays using L-929 fibroblasts and rabbit periosteal cells revealed a cytocompatible material able to support cellular attachment and proliferation. The interest in this field of research is rising, shifting toward the development of biological replacements able to mimic and regenerate the bone tissue itself, also in relation to extensive bone damage, which in the past, was only solved using autografts or allografts [156]. In Table 3, a selection of papers published in the last 10 years in this field is displayed with details in terms of collagen source, blending, post-production treatment, and application.

## 5. Surface Modification

Chemical, morphological, and biological requirements for currently used biomaterials strictly depend on their intended application. Other than characteristics such as non-toxicity, biocompatibility, structural and mechanical stability, and favorable degradation kinetics, a significant challenge in advancing biomaterials lies in enhancing the effectiveness of bioactivity by incorporating more intricate biological functionalities enabling effective communication with the environment. Tackling this challenge means drawing inspiration from biology and emulating biological properties or processes naturally already present in the body. As discussed in this review, the first task is to produce biocompatible materials characterized by “bulk properties” with enhanced properties to accomplish cell seedling and accompanying degradation over a reasonable time, sometimes provided with the incorporation of bioactive factors encapsulated within the biomaterial that are released over time to elicit a specific response or modulate tissue formation [167,168,169]. However, as soon as contact with a living organism takes place, the surface of biomaterials is the interface that is responsible for the very first interaction and communication process. Consequently, efforts have concentrated on adapting biomaterial surfaces in a way that they can improve cell attachment and selectively interact with specific cell receptors. This interaction is driven by molecular recognition events that trigger precise biological or cellular functions. The main strategies adopted for surface modification can be divided into physical surface modifications and chemical surface modifications.

### 5.1. Physical Surface Modifications

Owing to safety, cost-effectiveness, and biocompatibility, physical methods such as physical absorption, composite, and plasma, are sometimes preferred rather than chemical methods. Physical surface modifications, by inducing surface nano- or micro-patterning, have been demonstrated to have a significant influence on cell behaviors in terms of cell shapes and migration, protein synthesis, and gene expressions. This direct effect on the ability of cells to orient themselves, migrate, and produce organized cytoskeletal arrangements was demonstrated and discussed in the 1990s by Flemming et al. [170]. The majority of the studies used photolithography, through which a substrate is coated with a thin polymeric film exposed to light that irradiates only selected regions due to the presence of a mask. Irradiated regions are subjected to photochemical reactions that make them more soluble or less soluble in specific solvents, later used to develop either positive or negative tone images of the mask. The formation of surface microgrooves or ridges has shown significant control over cellular behaviors with specific cell alignments [171]. 

**Figure 5:**
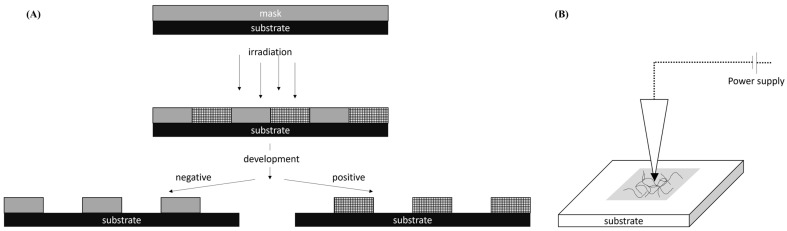
Schematic diagram of (**A**) the photolithographic process during the irradiation and development stages and (**B**) the electrospinning process for surface modification, and production of fine dispersed fibers from natural or synthetic polymers.

Pompo et al. studied the impact of coated electrospun poly(ε-caprolactone)(PCL) with hydroxyapatite with ionized jet deposition (IJD) on mesenchymal stem cell behavior. During electrospinning, a polymer solution is spun with the application of a tunable potential electric field to obtain polymeric fibers. Their result indicated that bone apatite coatings influence MSC early adhesion and spreading within the electrospun scaffolds, supporting better colonization [172]. The effect of plasma surface modification on electrospun polyhydroxybutyrate (PHB) mats was studied by Mohammadalipour et al., in which significant improvement in MG63 cell-scaffold interactions on modified nanofibers was shown [173]. To investigate the effect of nanocomposite scaffolds on cell behavior in bone tissue engineering, Akbari et al. fabricated poly-vinylidene fluoride (PVDF)/PCL/hydroxyapatite electrospun scaffolds. Their outcomes demonstrated that the three-component scaffold reveals significant improvement in cell adhesion and function compared with the PCL counterpart [174]. Figure 5A,B displays a schematic overview of the photolithographic and electrospinning processes for surface modification.

In terms of modern advancement, one of the most emerging techniques of the 21st century, 3D printing and, in particular, fused deposition modeling, is revolutionizing the field of surface modification of scaffolds, finely and sub-micrometrically tuning the surface properties in terms of geometry, roughness, etc. [175]. Indeed, 3D printed scaffolds display all the advantages of the past techniques already able to design specific geometries, with the major benefits of enhanced resolution and customizing the production in a patient-tailored way [176]. The implementation of 3D printing technology in the field of healthcare is connected not only to the advantages in terms of faster production and wasted material decrease but also to the possibility of exploiting printing precision with surface modification methods already known from the past. For example, Shi et al. proposed a promising approach to improve the surface bioactivity of polyetheretherketone (PEEK) implants using 3D printing technology. PEEK, due to its good biocompatibility and mechanical strength, is widely used as an orthopedic implant material, suffering, however, biological inertness. By a combination of fused deposition modeling and sulfonation treatment, they succeeded in promoting the formation of micropores on the surface of PEEK implants. Tests for adhesion and proliferation of stem cells from human exfoliated deciduous teeth indicated that the final material showed superior biocompatibility and osteogenic activity of phalanges exhibited significantly stronger osteogenic activity than the control groups [177]. A specific discussion of this technique will be given in a specific paragraph.

### 5.2. Chemical Surface Modifications

One of the essential considerations in designing engineered scaffolds is the use of materials that can impart a hydrophilic nature to their surface, given the inability of cells to adhere to hydrophobic surfaces and strongly limiting their ability to proliferate and differentiate. However, most of the synthetic polymers nowadays developed for biomedicine are mostly hydrophobic, hence lacking cell recognition sites with rather poor cell attachment to the scaffold [178].

Chemical surface modifications are used to immobilize biopolymers or biomolecules that can improve cell–substrate interactions. One of the chemical methods to modify the surface of the scaffold is the wet chemistry method. The wettability (hydrophobicity and hydrophilicity) of the substrate can affect surface protein adsorption and cell adhesion. Grigora et al. demonstrated that the hydrophilicity of a scaffold’s surface has a crucial effect on cell adhesion and proliferation [179]. They revealed that chemical modification of 3D-printed poly(lactic acid)/montmorillonite (PLA/MMT) with Strontium bioglass (SrBG) and SrBG with nanohydroxyapatite led to a decrease in the water contact angle of the scaffold’s surface and finally, cell adhesion. Since appropriate protein functionalization on biomaterial substrates is important for cell attachment, proliferation, and differentiation, Mou et al. functionalized electrospun silk fibroin membranes with laminin-511 [180]. In vitro studies showed that laminin coating of the silk fibroin membranes is essential for robust cell adhesion and podocyte differentiation in human stem cells. In another study, Moroni and co-workers studied the thiol-ene conjugation of a vascular endothelial growth factor (VEGF)-mimetic peptide to electrospun scaffolds for potential applications in angiogenesis [181]. They showed that conjugation of PCL-diacrylate (DA) fibrous scaffolds with the VEGF peptide not only increased the wettability of scaffolds but also induced phosphorylation of the VEGF receptor and enhanced human umbilical vein endothelial cell (HUVEC) survival, proliferation, and adhesion.

Another technique for chemical modification is the historical treatment with plasma, which introduces polar groups on the surface of the scaffold, increasing the hydrophilicity of the surface. Cold plasma, mostly used for temperature-sensitive polymers, is formed with gas ionization, producing a mixture of ions, atoms, electrons, and molecules due to the presence of a strong electric field [182]. No chemical agents or solvents are used, avoiding any risk of contamination or increase in scaffold toxicity. Laput and co-workers investigated low-temperature arc-discharge plasma in a nitrogen atmosphere on PLA scaffolds. They induced surface carbonization, accompanied by a carbon atomic concentration increase, influencing PLA wettability characteristics. Increasing nitrogen atomic concentration resulted in a decrease in the water contact angle. The viability of macrophages was superior to the control level when PLA scaffolds were treated with plasma, explained by the highest content of -C-N bonds and the optimal value of the surface energy ensuring stable cell adhesion and satisfactory conditions for cell growth [183]. Namhongsa et al. recently developed a two-component composite scaffold consisting of a poly(L-lactide-co-ε-caprolactone) 3D-printed component, providing mechanical strength, and a poly(L-lactide-co-glycolide)-based electrospun fiber component, acting as a trigger for the extracellular matrix to improve cell–substrate interactions. A nitrogen–argon plasma treatment was performed to enhance the surface properties, demonstrating a great reduction in the water contact angle. Cells cultivated on the final scaffolds displayed a significant increase in attachment and proliferation and a higher presence of healthy cells when compared with untreated ones [184].

Another type of chemical surface modification is directly connected to the use of proteins. Efforts in creating biomimetic surfaces, together with displaying adequate surface wettability, have been accompanied by the focus on the presence of the coatings of naturally occurring ECM proteins like fibronectin, vitronectin, collagen, and laminin. However, there are several limitations associated with the utilization of long-chain proteins derived from the ECM. Even if purified and processed before use, being derived from animal sources, proteins may still elicit immunological responses with associated risk of infections. Moreover, during immobilization on scaffold surfaces, large proteins will only have part of their structure in the opportune orientation for cellular interaction [185]. In addition, due to insolubility or favored kinetics of desorption from the material surface, proteins might encounter rapid elimination from the body, also in consequence of naturally occurring enzymes [186]. For all these reasons, the use of short peptide sequences is preferable in many avenues of research. Smaller oligopeptides offer enhanced cost-efficiency and stability when subjected to sterilization or storage. The absence of a 3D structure avoids any concern related to protein denaturation and inaccessibility of cell-binding sites. In addition, oligopeptides can be chemically synthesized to maximize targeted interaction with specific cell types or adhesion receptors. Furthermore, due to their compact size, oligopeptide fragments can be more densely immobilized on material surfaces, enhancing their bioactivity. One of the most frequently used oligopeptide in biomimetic surface modification is the tripeptide RGD, the cell signaling domain derived from fibronectin and laminin. Thijssen and co-workers recently published a single-step strategy toward cell-adhesive polymer networks where thiol-ene chemistry was used to achieve alkene-functionalized polycaprolactone (PCL) crosslinking. Moreover, using a cysteine coupling site, the cell-binding motif C(-linker-)RGD was covalently bound throughout the PCL networks during crosslinking. Human foreskin fibroblasts were cultured on the functionalized PCL networks and a clear positive effect of the modification on the cell adhesiveness of the PCL networks was evidenced [187]. 

In general, anchoring bioactive oligopeptides requires the formation of covalent bonds with the biomaterial surface. Nonetheless, this immobilization strategy implies the existence of reactive surface groups. To address this issue, scientists have developed various strategies to generate functional groups on biomaterial surfaces, such as the introduction of active functional groups by synthesizing novel graft copolymers with desired reactive groups [188].

Recently, many groups have designed polymer coatings, grafts, and protein/cell-based coatings to develop biomaterials that take advantage of existing biological processes and prevent immune recognition. In fact, given that biomimetic materials are synthetic versions of naturally occurring structures, the biocompatibility and cell response and functions of these designs must be considered when implanting or introducing devices in vivo. In terms of biocompatibility, the body as a host is excellent at recognizing and repelling non-self-objects through the foreign body response. Non-specific protein adsorption (NPA) on implants is the first step of this process, and it must be mitigated to ensure the longevity of implants [189]. Other therapeutic delivery approaches also face the barrier of the phagocytic cells of the innate immune system (macrophages), which are central for clearing foreign bodies recognized in various tissues [190]. Thus, including inert or biofunctionalized materials in biomedical technologies is essential. Biofouling, the accumulation of proteins and other entities that render implants nonfunctional or structurally deficient, can be prevented by coating base implants in polymer solutions. Fukuda and Xu utilized a biomimetic hydrosilane-functionalized 2-methacryloyloxyethyl phosphorylcholine (MPC) to coat the channels of a nanofluidic device and reduce the adhesion of bovine serum albumin (BSA), hemoglobin (Hb), and cytrocrome *c* [191]. They found that increasing the concentration of phosphorylcholine head groups on the channel walls increased the reduction rate. As anionic, neutrally charged, and cationic proteins were tested on their device, the MPC coating may be used to prevent NPA from many other proteins without disrupting fluid flow through the device. Here, tris(penta-fluorophenyl)borane (TPFB) was added to the coating solution to act as a catalyst, though other chemical coating methods like grafting do not always require molecular catalysts. Polymer grafting is triggered by a polymerization reaction on treated surfaces to spontaneously generate long polymer chains and can be accomplished using gamma irradiation, which efficiently creates free radicals to tether polymers to a target surface [192]. Jeong et al. utilized gamma rays to graft zwitterionic MPC on a para-toluene sulfonate-doped polypyrrole electrode, which does not have covalent bonds and, therefore, cannot be covalently bound to MP, to introduce anti-biofouling properties to the electrode without interfering with its electrochemical properties [193]. Thus, the authors circumvented the issue of protein adherence to sustain the biofunctionality of the polypyrrole (pPy) electrode, which is used to electrically communicate with biological systems. Indeed, biolectrodes, including metallic and conductive polymers, have the disadvantage of biofouling by bacterial contamination in biological systems. 

Alternatively, proteins and other biomolecules may be covalently attached with sophisticated techniques to the surfaces of implants and particles to increase their biocompatibility and functionalize them for proliferation regulation and recognition of cell-surface proteins. Liu et al. covalently bound laminin and heparin to an alkali-treated, amino-rich, microporous titanium-based plate and immersed the plate in an SDF-1 solution and found that the coating inhibited smooth muscle proliferation and promoted endothelial adhesions and proliferation [194]. This plate modeled a potential surface modification for titanium vascular stents that improved biocompatibility and may prevent intimal hyperplasia.

Other biomimetic designs utilize imprints of existing biological structures in addition to covalent conjugation of proteins to the surfaces of substrates and particles. Lithography is a common method for microfabricating micron-scale structures and some of the nanoscale details on the master template [195]. For example, soft lithography is used to create imprints/stamps in elastomeric polymers by pouring polymer onto a mold made from a master template [195], while electron-beam lithography faithfully fabricates patterns on the electron-sensitive substrate with a focused beam of electrons [196]. Once the initial surface modification is complete, biomolecules can be immobilized to the surface. Nguyen et al. used electron-beam lithography to create a silicon master template with discoidal cavities similar to a red blood cell and then applied soft lithography to mold polydimethylsiloxane (PDMS) films into the discoidal shape [197]. The particles were then coated with red blood cell membranes with sonication, which prevented their internalization by macrophages and improved the particles’ biocompatibility, blood circulation times, and half-lives compared with uncoated particles. Lu and co-workers used a soft lithography technique to create an imprint mold of human breast cancer cell lines, MCF-7, circulating on PDMS, which was then functionalized with biotinylated natural antibodies anti-EpCAM to improve recognition and capture of target cells [198]. The biocompatibility of the imprint was demonstrated by measuring the cell viability of the captured cells, resulting in new prospects for cell–material interaction interfaces for biological applications.

## 6. 2D and 3D Fabrication Techniques

Developments in fabricating technologies at the micro- and nano-scale and 2D and 3D techniques paved the way for novel cell–substrate interaction studies. The production of medical devices made of nanoscale fibers and pores with a higher specific surface area has shown a significant improvement in cell–substrate interactions. Many groups studied the influence of the fiber diameter of electrospun substrates on NSC and fibroblast cell behavior [199,200]. Their results proved the positive impact of nano-size fibers (thinner fibers and higher specific area) on cell adhesion, proliferation, and differentiation. The 3D bioprinting technique enables the fabrication of complex geometries, porous structures, interconnected networks, and precise cell positioning for 3D cell culture. It can adequately mimic conditions in vivo and the complex cell–substrate interaction in all three dimensions, which promotes cell adhesion. Ozbolat et al. demonstrated that the 3D printing of PDMS facilitates the adhesion of breast cancer cells, whereas cast samples do not allow cellular adhesion without the use of additional coatings such as ECM proteins [201]. Other researchers reported the use of 3D bioprinted gelatin–sodium alginate/rat Schwann cell scaffolds with the promotion of cell viability, proliferation, and adhesion [202]. 

Overall, while mimicking native microenvironments has been significantly developed thanks to new fabrication techniques, physical and chemical modification of the biological environment is, nevertheless, necessary to provide acceptable conditions.

### Advances and Limitations in 3D Bioprinting

**Figure 6:**
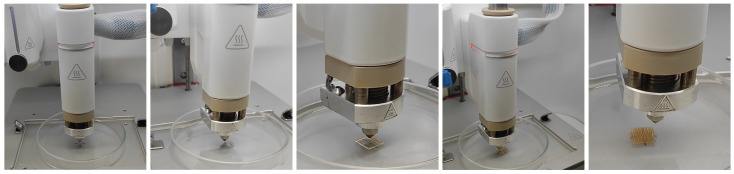
Representation of a thermoplastic cartridge under working conditions while printing PLA-modified scaffolds, courtesy of Marzorati.

**Figure 7:**
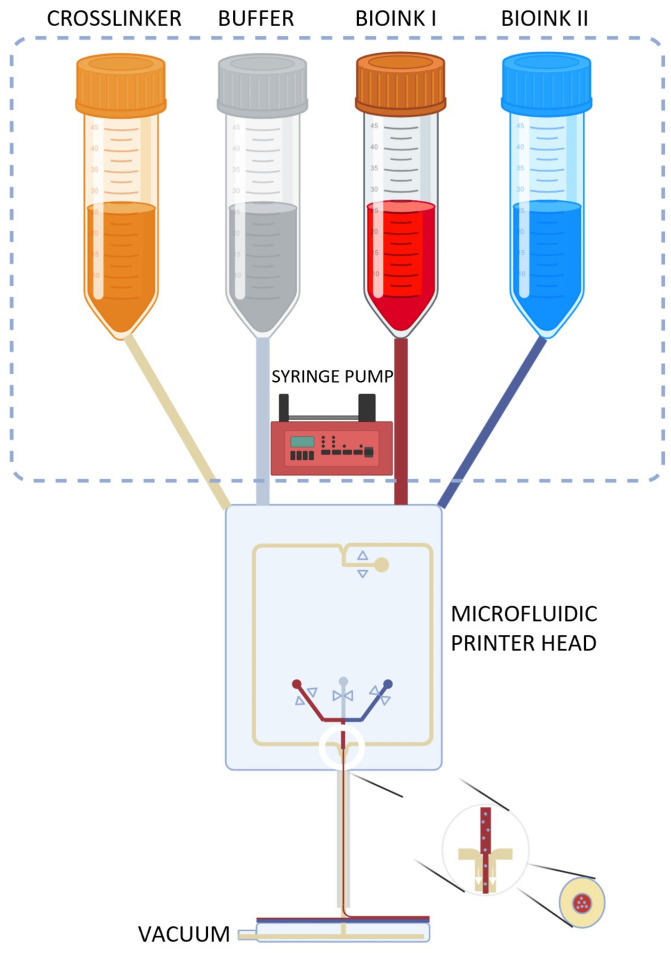
Microfluidic bioprinting process, which coaxially combines bioinks with a crosslinker sheath fluid to form fibers deposited into 3D structures. In this technique, the integration of microfluidic systems with traditional extrusion-based bioprinting facilitates tuning the structural and compositional properties of tissue constructs during printing. Microfluidic fabrication techniques enable the control of minute amounts of liquids, cells, and molecules.

A 3D bioprinter (Figure 6) is a specialized type of 3D printer capable of creating three-dimensional biological structures by layering biological materials, such as cells, tissues, and biomaterials, in a precise and controlled manner. This technology holds great promise for various applications in the fields of regenerative medicine, tissue engineering, drug testing, and personalized medicine [203]. The favorable outcome of bioprinted models closely relies on the use of appropriate biomaterial ink/bioinks with optimal printability, high biocompatibility, and bioactivity and bioprinting techniques with high spatial resolution and harmless for cells (Figure 7) [204].

In recent years, 3D-bioprinting techniques have witnessed significant development thanks to advanced technology that has enabled them to design systems with better resolution, better cost-effectiveness, higher printing speed, and less damage to cells. In addition, these new techniques have been developed to allow the creation of more complex and intricate structures with a high spatial resolution that can better recapitulate in vivo human biology.

New formulations of bioink have been one of the most exciting research topics within 3D bioprinting technology, which are crucial for the progress of 3D bioprinting as they directly impact the quality, functionality, and applicability of bioprinted tissues and organs. Research in biomaterials has focused primarily on studying polymers to obtain bioinks with mechanical, chemical, and biological characteristics suitable for each application [205].

A key component of bioink is the formulation with cells. Studies focused on cell types applied to 3D bioprinting represent a major part of all publications with a significant focus on studying the viability, differentiation, and maturation of different types of stem cells (e.g., MSC, induced pluripotent stem cells, adipose-derived stem cells). Spheroids and, more recently, organoids have also been used in bioprinting and have gained attention in the last few years, presenting exciting developments for translational medicine and 3D-disease modeling [205].

In addition, to the introduction of new natural and synthetic biomaterials for 3D bioprinting, new formulations based on adding bioactive nanoparticles to bioinks and using smart biomaterial as bioinks have been the new research fields in recent years.

Nanocomposite bioinks are a type of bioink used in 3D bioprinting that incorporates nanoparticles or nanomaterials into the bioink formulation to create hybrid bioinks. These nanoparticles can be made from various materials, including metals, polymers, ceramics, and carbon-based materials like graphene or carbon nanotubes. The addition of nanoparticles to bioinks imparts unique properties and functionalities to the resulting printed structures. Nadernezhad et al. demonstrated that agarose and Laponite nanosilicates not only developed nanocomposite shear thinning of nanocomposite bioinks for extrusion 3D bioprinting applications but also significantly improved the bioactivity of nanocomposite hydrogels with increased metabolic activity of encapsulated cells and the ability of cells to extend and change their morphology [206]. Alarçin et al. optimized the printability of methacrylated gelatin (gelatin methacrytoyl, GelMA) with layered double hydroxides (LDHs) [207]. Their results indicated that the addition of LDHs into the GelMA network could improve the physical and biological properties of the nanocomposite scaffolds. The addition of LDHs led to continuous printing of stable structures with well-defined geometry, shape fidelity, and enhanced mechanical properties, while osteoblasts encapsulated in the nanocomposite bioinks without grow factor exhibited high cellular viability, spreading, and proliferation.

In the context of 4D bioprinting, a 4D bioink would refer to a material that can be 3D bioprinted and then undergoes time-dependent changes in response to various triggers or environmental cues. These materials can change their shape, structure, or function in response to external stimuli such as temperature, pH, humidity, light, or specific chemicals [208]. Pedro et al. reported a 4D biofabrication method for cartilage engineering based on the differential swelling of a smart multi-material system made of tyramine-functionalized hyaluronan and alginate [209]. In their study, a novel smart multi-material system was developed for 4D bioprinting based on the shape change transformation of 3D scaffolds into curved structures that were capable of maintaining high cell viability and allowed for hMSC-derived cartilaginous matrix deposition. Kitana et al. demonstrated the possibility of the fabrication of hollow tubular structures in scalable diameter with controlled shape transformation of 3D-printed hydrogel structures using oxidized alginate-gel as a shape-changing biomaterial [210]. Additionally, HUVECs showed appropriate growth, adhesion, high average cell viability, and proliferation on the printed structures.

An interesting and attractive technology is microfluidics, which deals with the manipulation and control of tiny volumes of fluids (typically at the microliter or nanoliter scale) in microchannels [211]. Combining extrusion-based 3D bioprinting with microfluidic systems leads to the control of minute amounts of liquids, cells, and molecules precisely and creates more complex and functional tissue constructs with integrated vasculature for better nutrient and waste exchange. It was demonstrated in several studies that using a microfluidic 3D bioprinter causes well-positioned cells and high-resolution 3D models [212,213]. Wang et al. successfully printed small-diameter vascular conduits with tough hydrogel bioinks consisting of gelatin and alginate with suitable rheological properties and cell-benign crosslinking using microfluidic bioprinting [213]. Yin et al. utilized a microfluidics-based coaxial bioprinter with alginate as a model biomaterial for generating refined hydrogel structures with dimensions and morphologies inspired by salivary epithelia [214]. They demonstrated well-printed fibers and hollow tubes in a broad range of accessible structure sizes and successful preservation of primary human salivary gland cells viability for bioprinted tubes.

Digital light processing (DLP)-based 3D bioprinting is a 3D printing technology that utilizes digital light projection to selectively solidify layers of photosensitive biomaterials, creating complex 3D biological structures. DLP-based 3D bioprinting has emerged as a promising biofabrication technique due to its high resolution, high cell viability, and rapid speed [215]. A DLP 3D printer can use either liquid crystal display (LCD) or digital micromirror device (DMD) technology in conjunction with DLP technology to create high-resolution 3D prints. The LCD panel consists of an array of pixels that can be individually controlled to allow or block light, while the DMD technology uses an array of microscopic mirrors (typically in the millions) to selectively reflect or block UV light. DLP 3D bioprinters using LCD and MDM technologies offer high resolution, speed, and the ability to create detailed and intricate biological 3D models.

Rajput et al. used photocurable methacrylated silk fibroin as a bioink for DLP-based 3D bioprinting to fabricate complex structures, including microchannels, and vascularized bone-like scaffolds with high precision and good fidelity bioprinted hydrogels showed rheological and mechanical properties characteristic of human tissues [216]. Also, the bioprinted hydrogels revealed good cytocompatibility and proliferation and induced osteogenic differentiation of pre-osteoblasts even in the absence of soluble factors.

Kumari et al. synthesized a photocurable polysaccharide and used it to fabricate tissue scaffolds using DLP-based 3D printing technology [217]. The bioprinted methacrylate-κ-carrageenan (MA-κ-CA) hydrogels exhibited a good combination of viscoelastic and mechanical properties required for soft tissue engineering. Fabricated NIH 3T3 cell-laden hydrogels demonstrated high cell viability and supported proliferation over 30 days.

Overall, 3D bioprinting holds immense promise for regenerative medicine, tissue engineering, and personalized healthcare, and significant advances have occurred in the field of 3D bioprinting; however, it faces several challenges that need to be addressed to unlock its full potential. Some of the challenges in 3D bioprinting include bioprinting at scale, bioink development, immune response, long-term safety and efficacy, multi-cell type printing, complex tissue structures, and cost and accessibility. Despite these challenges, researchers and scientists continue to make significant strides in 3D bioprinting, and the field holds great promise for revolutionizing healthcare and regenerative medicine. Overcoming these challenges will require interdisciplinary collaboration, technological innovation, and ongoing research efforts.

## 7. Artificial Intelligence (AI) Implications and Future Perspective

Biomimetic intelligence and humanoid robots are emerging to be applied in different complex scenarios (e.g., living systems, as well as home environments) with biological functions [218]. Biomimetic AI is exciting researchers due to its impact on our lives. This field focuses on the developments of algorithms inspired by biological working mechanisms such as, for example, the genetic algorithm [219] and ant colony optimization [220]. Recently, advances in biomimetic intelligence achieved tremendous results: biomimetic sensors and sensing techniques [218] and evolution-inspired algorithms [221,222,223,224] have developed an intuition similar to that used by scientists [225,226]. The acceleration of science due to AI is in continued transformation, but bionics and biomimicry from AI are not yet really transferred to human applications. To date, the major limitations are in reproducing accurate data on a large scale. In the field of biomaterial design, researchers fight with an enormous number of experiments, models, predictions, and failures, due to complex and dynamic environments to emulate and thus AI may serve as a silent ally. The combination of machine learning and AI is to be adjusted, as machine learning imposes restrictions on AI [227]. Recently, a hybrid-active learning AI algorithm was reported to integrate machine learning with density-functional theory, thermodynamic calculations, and experiments [228]. Another hybrid approach was described by Vecchio and co-workers using machine learning and density functional theory calculations. The validated protocol was able to predict the entropy formed by 70 new compositions [229]. Chat generative pre-trained transformer (ChatGPT, OpenAI) and GPT-4 (the latest version of ChatGPT), as subgroups of AI, are able to elaborate information (for example, from the internet) to produce responses, such as the human language (although still with errors). Similarly, the use of ChatGPT may serve to address troubleshooting in 3D printing, including the control of the head and printer extruder, the optimization of the final product, the material, the ink, and the time spent [230]. By reading from machine learning, ChatGPT can improve its abilities and optimize the Gcode, thus providing insights for additive manufacturing. In recent years, there have been advances in AI for biology, engineering and biomedical engineering, environmental science, and ecology [231,232]. Recently, Cheng et al. described the exploration of ChatGPT-4 in biomedical engineering for the development of new biomimetic materials with the generation of existing data and knowledge, by simulating biological systems, designing experiments, and identifying patterns not identified by human researchers [232]. Nevertheless, given the great potential of ChatGPT, several shortcomings need to be addressed, starting from a conscious use.

In conclusion, in this non-exhaustive review, the authors presented the current state and the complexity of resembling a living system or a part of it. Bioinspiration and biomimetic strategies are providing an advancement in materials for in vivo design. The recent progress in 3D printing will provide to introduce cells and biological functions starting from a biocompatible ink. Other non-discussed points, such as the synthesis of artificially biomimetic membranes [233], growth factor mimicking-peptides [234], chemotactic signals, and electrical stimuli [235] are important aspects to examine in depth. 

Certainly, the open question is to be able to grasp the enormous potential offered by biological systems to address medical and societal needs with a multidisciplinary approach. Despite significant remaining challenges, biomimetic materials are reliable scaffolds currently promising for a wide range of applications, including tissue engineering, genome technologies, imaging, and drug delivery.

Recently, the literature reported the clinical statute of bioinspired and biomimetic materials [236,237], in particular, in wound healing and tissue regeneration. As growth factor and cytokine administrations are under clinical evaluation, future studies may include growth factors in constructed materials for promising improved results in wounds. Mineralized collagen is under clinical investigation in orthopedics, stomatology, and neurosurgery [238,239]. More than 100 studies are underway to investigate the efficiency and safety of chitosan and its related biomaterials in clinical use, from dental and bone applications to treating hemorrhage and wounds [240]. 

A crucial point to evaluate regards the biological safety of biomaterials. A good direction should focus deeper on the selection of safe materials and modifiers: this step is not always carried out as many crosslinkers are toxic and immunogen. Moreover, different categories of biological safety must be considered, including systemic toxicity, local toxicity, carcinogenicity, and central nervous system damage. To date, scientists and governments have not yet provided a well-documented plan to ensure biological safety in the use of biomaterials.

## Figures and Tables

**Table 1:** Elastic moduli, *E*, of tissues and materials.

Tissue	Young’s Elastic Modulus	Reference
Human epithelial cells (normal)	1.60 kPa	[14]
Human epithelial cells (cancer)	1.40 kPa	[14]
Human platelets	1–50 kPa	[15]
Kidney	180 kPa	[16]
Skin (different species)	20–40 Mpa	[17]
Muscle	480 Mpa	[18]
Tendon	43–1660 Mpa	[19]
Elastin-free tendon	1.2 Gpa	[20]
Cartilage	100–500 kPa	[21]
Human proximal tibia	11–14 Gpa	[22]
Biomaterial	Young’s Elastic Modulus	Ref.
Gelatin (Ge) ^1^	0 Pa < *E* < 75 kPa	[23]
Collagen mimetic peptide	~8 kPa	[24]
SAPs	0.1 kPa < *E* < 10 kPa	[25]
Hyaluronic acid (HA) hydrogel ^2^	1 kPa	[26]
HA-Ge	22 kPa	[27]
Fmoc-Phe-Phe-OH	200 kPa	[28]
Alginate hydrogel ^3^	117 kPa	[29]
Cross-SAPs	200 kPa < *E* < 850 kPa	[25,30]
Chitosan	~ 7 Mpa	[31]
Amphiphile polymer conetworks	5 Mpa < *E* < 200 Mpa	[32]
H-Phe-Phe-OH nanotubes	20 Gpa	[28]
Gelatin methacryloyl (GelMA)	3 kPa < *E* < 184 kPa	[33]
Collagen type I (in rat)	5–11.5 Gpa	[34]
Collagen (mammalian tendon)	1.2 Gpa	[12]

^1^ Measured at 20 °C (gelatin is very sensitive to the temperature); 0 Pa means that the solution is simply liquid at low concentration. ^2^ HA crosslinked with 5% *w*/*t* 1,4-butanediol diglycidyl ether (BDDE)**.** ^3^ Alginate hydrogel crosslinked with Ca^2+.^

**Table 2:** Classification of hydrogels.

Class	Subclass	Examples	Reference
Source	Natural	hyaluronic acid chitosan alginate	[76] [77] [78]
	Synthetic	poly (vinyl alcohol) (PVA) poly (ethylene glycol) (PEG) poly (acrylic acid) (PAA)	[78] [78] [79]
Charge			
	Ionic		
	Cationic	ε-poly-l-lysine (EPL) with poly(ethylene glycol) diglycidyl ether) (PEGDGE)	[80]
	Anionic	poly(oligo ethylene glycol monoacrylate-*co*-acrylic acid)	[81]
	Non-ionic	hydroxyethyl cellulose	[82]
	Zwitterionic	poly(sulfobetaine methacrylate)	[83]
Composition			
	Homopolymeric	poly(*N*-isopropylacrylamide)	[84]
	Heteropolymeric (co-polymeric/multi-polymeric)	oligo(ethylene glycol) with di(meth)acrylate	[85,86]
Physical properties			
	Solid	β-hairpin peptide-based	[87]
	Liquid	gelators with ion liquids	[88]
	Mucoadhesive	polymer with intestinal mucus	[89]
Crosslinking mechanism			
	Physical	polyelectrolyte hydrogels ionizing radiation MW irradiation	[90] [52,91] [57]
	Chemical	with small molecules	[54,92,93]

**Table 3:** Details on extraction methods and pre/post-treatments for the development of collagen-based biomaterials in the recent literature.

Collagen Source	Blending Procedure	Scaffold Production Technique	Stabilization Treatment	Application	Authors	References
Bovine, porcine, and ovine tendons	Pure collagen	Lyophilization	Crosslinking in a 10 mM EDC and 5 mM NHS	Tissue engineering application	Ghodbane et al.	[144]
Type I bovine collagen	Pure collagen	3D printing	Fiber formation buffer solution + crosslinking in EDC in 90% acetone	Tissue engineering application	Nayak et al.	[145]
Femoral condyles old bovids	Pure collagen	3D printing	Riboflavin crosslinking	Natural-mimicking hydrogels	Diamantides et al.	[149]
Type I bovine collagen	Pure collagen	Lyophilization	Collagen methacrylamide + crosslinking in EDC/NHS	Tissue engineering application as dynamic, photoactive hydrogel	Gaudet et al.	[150]
JellaGel™ solution, jellyfish collagen	Pure collagen	Gel formation	Genipin crosslinking	Cartilage regeneration	Riacci et al.	[93]
Sea urchin waste	Pure collagen	Lyophilization	UV crosslinking	Wound healing	Ferrario et al.	[151,152]
*Catostylus mosaicus* jellyfish	Collagen/agarose	Gel formation	None	Tissue regeneration	Rastian et al.	[158]
Dried squid and dried jellyfish	Pure collagen	Lyophilization	None	Tissue regeneration	Jankangram et al.	[159]
*R. esculentum* jellyfish	Pure collagen	Lyophilization	Crosslinking in EDC/NHS	Hemostatic applications	Xiaochen et al.	[160]
*Doryteuthis singhalensis* squid waste	Pure collagen	Gel formation	None	Tissue regeneration	Veeruraj et al.	[161]
Type I collagen from equine tendon	Hydroxyapatite/collagen	Gel formation, biomineralization	Ribose crosslinking	Bone and osteochondral regeneration	Dellaquila et al.	[162]
Commercial Type I collagen-based recombinant peptide	Fe-doped Hydroxyapatite/collagen	Water-in-oil emulsification process	None	Bone-like microspheres with intrinsic magnetic properties	Fernandes Patrício et al.	[163]
Type I collagen from rat tails	Fe and Mn-doped Hydroxyapatite/Collagen	Lyophilization	None	Bone regeneration	Yu et al.	[164]
Bovine tendon collagen	Chitin–hydroxyapatite–collagen	Lyophilization	Epichlorohydrin crosslinking	Bone regeneration	Xing et al.	[165]
Bovine tendon collagen	Zinc silicate/nano-Hydroxyapatite/collagen	3D printing	None	Angiogenesis and bone regeneration	Yue et al.	[166]

## References

[1] Naik R.R., Singamaneni S. (2017). Introduction: Bioinspired and Biomimetic Materials. Chem. Rev..

[2] Rowley T. (2013). Science imitates life. Lab Anim..

[3] Glaser D.E., Viney C. (2013). Biomimetic Materials. Biomaterials Science.

[4] Atala A., Kasper F.K., Mikos A.G. (2012). Engineering Complex Tissues. Sci. Transl. Med..

[5] Lavik E.B., Zheng G. (2018). Biomimetic Materials. Bioconjug. Chem..

[6] Liebschner M., Bucklen B., Wettergreen M. (2005). Mechanical Aspects of Tissue Engineering. Semin. Plast. Surg..

[7] Mantha S., Pillai S., Khayambashi P., Upadhyay A., Zhang Y., Tao O., Pham H.M., Tran S.D. (2019). Smart Hydrogels in Tissue Engineering and Regenerative Medicine. Materials.

[8] Koutsopoulos S. (2016). Self-assembling peptide nanofiber hydrogels in tissue engineering and regenerative medicine: Progress, design guidelines, and applications. J. Biomed. Mater. Res. Part A.

[9] Thomson J.A. (1968). On Growth and Form.

[10] Currey J.D. (2002). Bones: Structure and Mechanics.

[11] Vincent J.F.V. Structural Biomaterials.

[12] Muiznieks L.D., Keeley F.W. (2013). Molecular assembly and mechanical properties of the extracellular matrix: A fibrous protein perspective. Biochim. Biophys. Acta Mol. Basis Dis..

[13] Coenen A.M.J., Bernaerts K.V., Harings J.A.W., Jockenhoevel S., Ghazanfari S. (2018). Elastic materials for tissue engineering applications: Natural, synthetic, and hybrid polymers. Acta Biomater..

[14] Guz N., Dokukin M., Kalaparthi V., Sokolov I. (2014). If Cell Mechanics Can Be Described by Elastic Modulus: Study of Different Models and Probes Used in Indentation Experiments. Biophys. J..

[15] Radmacher M., Fritz M., Kacher C.M., Cleveland J.P., Hansma P.K. (1996). Measuring the viscoelastic properties of human platelets with the atomic force microscope. Biophys. J..

[16] Karimi A., Shojaei A. (2017). Measurement of the Mechanical Properties of the Human Kidney. IRBM.

[17] Vogel H. (1980). Influence of maturation and aging on mechanical and biochemical properties of connective tissue in rats. Mech. Ageing Dev..

[18] Buchanan C.I., Marsh R.L. (2001). Effects of long-term exercise on the biomechanical properties of the Achilles tendon of guinea fowl. J. Appl. Physiol..

[19] Wren T.A., Yerby S.A., Beaupré G.S., Carter D.R. (2001). Mechanical properties of the human achilles tendon. Clin. Biomech..

[20] Gosline J., Lillie M., Carrington E., Guerette P., Ortlepp C., Savage K. (2002). Elastic proteins: Biological roles and mechanical properties. Philos. Trans. R. Soc. Lond. Ser. B Biol. Sci..

[21] Varanko A.K., Su J.C., Chilkoti A. (2020). Elastin-Like Polypeptides for Biomedical Applications. Annu. Rev. Biomed. Eng..

[22] Wu D., Isaksson P., Ferguson S.J., Persson C. (2018). Young’s modulus of trabecular bone at the tissue level: A review. Acta Biomater..

[23] Taberlet N., Ferrand J., Camus É., Lachaud L., Plihon N. (2017). How tall can gelatin towers be? An introduction to elasticity and buckling. Am. J. Phys..

[24] Parmar P.A., Chow L.W., St-Pierre J.-P., Horejs C.-M., Peng Y.Y., Werkmeister J.A., Ramshaw J.A.M., Stevens M.M. (2015). Collagen-mimetic peptide-modifiable hydrogels for articular cartilage regeneration. Biomaterials.

[25] Ciulla M.G., Pugliese R., Gelain F. (2022). Boosted Cross-Linking and Characterization of High-Performing Self-Assembling Peptides. Nanomaterials.

[26] Kaya G., Oytun F. (2020). Rheological Properties of İnjectable Hyaluronic Acid Hydrogels for Soft Tissue Engineering Applications. Biointerface Res. Appl. Chem..

[27] Heris H.K., Rahmat M., Mongeau L. (2012). Characterization of a Hierarchical Network of Hyaluronic Acid/Gelatin Composite for use as a Smart Injectable Biomaterial. Macromol. Biosci..

[28] Adler-Abramovich L., Gazit E. (2014). The physical properties of supramolecular peptide assemblies: From building block association to technological applications. Chem. Soc. Rev..

[29] Malektaj H., Drozdov A.D., deClaville Christiansen J. (2023). Mechanical Properties of Alginate Hydrogels Cross-Linked with Multivalent Cations. Polymers.

[30] Pugliese R., Marchini A., Saracino G.A.A., Zuckermann R.N., Gelain F. (2018). Cross-linked self-assembling peptide scaffolds. Nano Res..

[31] Le H.R., Qu S., Mackay R.E., Rothwell R. (2012). Fabrication and mechanical properties of chitosan composite membrane containing hydroxyapatite particles. J. Adv. Ceram..

[32] Velasquez S.T.R., Jang D., Jenkins P., Liu P., Yang L., Korley L.T.J., Bruns N. (2022). Peptide-Reinforced Amphiphilic Polymer Conetworks. Adv. Funct. Mater..

[33] Wu Y., Xiang Y., Fang J., Li X., Lin Z., Dai G., Yin J., Wei P., Zhang D. (2019). The influence of the stiffness of GelMA substrate on the outgrowth of PC12 cells. Biosci. Rep..

[34] Wenger M.P.E., Bozec L., Horton M.A., Mesquida P. (2007). Mechanical Properties of Collagen Fibrils. Biophys. J..

[35] Hoeve C.A.J., Flory P.J. (1974). The elastic properties of elastin. Biopolymers.

[36] Trębacz H., Barzycka A. (2023). Mechanical Properties and Functions of Elastin: An Overview. Biomolecules.

[37] Saitow C.B., Wise S.G., Weiss A.S., Castellot J.J., Kaplan D.L. (2013). Elastin biology and tissue engineering with adult cells. Biomol. Concepts.

[38] Daamen W., Veerkamp J., Vanhest J., Vankuppevelt T. (2007). Elastin as a biomaterial for tissue engineering. Biomaterials.

[39] Aaron B.B., Gosline J.M. (1981). Elastin as a random-network elastomer: A mechanical and optical analysis of single elastin fibers. Biopolymers.

[40] Debelle L., Tamburro A.M. (1999). Elastin: Molecular description and function. Int. J. Biochem. Cell Biol..

[41] Li B., Alonso D.O.V., Bennion B.J., Daggett V. (2001). Hydrophobic Hydration Is an Important Source of Elasticity in Elastin-Based Biopolymers. J. Am. Chem. Soc..

[42] Luo T., Kiick K.L. (2013). Collagen-like peptides and peptide–polymer conjugates in the design of assembled materials. Eur. Polym. J..

[43] Ciulla M.G., Civera M., Sattin S., Kumar K. (2023). Nature-inspired and medicinally relevant short peptides. Explor. Drug Sci..

[44] Zhao B., Li N.K., Yingling Y.G., Hall C.K. (2016). LCST Behavior is Manifested in a Single Molecule: Elastin-Like polypeptide (VPGVG) n. Biomacromolecules.

[45] Betre H., Ong S.R., Guilak F., Chilkoti A., Fermor B., Setton L.A. (2006). Chondrocytic differentiation of human adipose-derived adult stem cells in elastin-like polypeptide. Biomaterials.

[46] Trabbic-Carlson K., Setton L.A., Chilkoti A. (2003). Swelling and Mechanical Behaviors of Chemically Cross-Linked Hydrogels of Elastin-like Polypeptides. Biomacromolecules.

[47] Lim D.W., Nettles D.L., Setton L.A., Chilkoti A. (2007). Rapid Cross-Linking of Elastin-like Polypeptides with (Hydroxymethyl)phosphines in Aqueous Solution. Biomacromolecules.

[48] Hrabchak C., Rouleau J., Moss I., Woodhouse K., Akens M., Bellingham C., Keeley F., Dennis M., Yee A. (2010). Assessment of biocompatibility and initial evaluation of genipin cross-linked elastin-like polypeptides in the treatment of an osteochondral knee defect in rabbits. Acta Biomater..

[49] Mozhdehi D., Luginbuhl K.M., Simon J.R., Dzuricky M., Berger R., Varol H.S., Huang F.C., Buehne K.L., Mayne N.R., Weitzhandler I. (2018). Genetically encoded lipid–polypeptide hybrid biomaterials that exhibit temperature-triggered hierarchical self-assembly. Nat. Chem..

[50] Pugliese R., Maleki M., Zuckermann R.N., Gelain F. (2019). Self-assembling peptides cross-linked with genipin: Resilient hydrogels and self-standing electrospun scaffolds for tissue engineering applications. Biomater. Sci..

[51] Pugliese R., Fontana F., Marchini A., Gelain F. (2018). Branched peptides integrate into self-assembled nanostructures and enhance biomechanics of peptidic hydrogels. Acta Biomater..

[52] Pugliese R., Montuori M., Gelain F. (2022). Bioinspired photo-crosslinkable self-assembling peptides with pH-switchable “on–off” luminescence. Nanoscale Adv..

[53] Chronopoulou L., Margheritelli S., Toumia Y., Paradossi G., Bordi F., Sennato S., Palocci C. (2015). Biosynthesis and Characterization of Cross-Linked Fmoc Peptide-Based Hydrogels for Drug Delivery Applications. Gels.

[54] Chronopoulou L., Toumia Y., Cerroni B., Pandolfi D., Paradossi G., Palocci C. (2017). Biofabrication of genipin-crosslinked peptide hydrogels and their use in the controlled delivery of naproxen. New Biotechnol..

[55] Pugliese R. (2022). Supramolecular-Covalent Peptides Self-Assembly: From Design to Regenerative Medicine and Beyond. Biophysica.

[56] Gaar J., Naffa R., Brimble M. (2020). Enzymatic and non-enzymatic crosslinks found in collagen and elastin and their chemical synthesis. Org. Chem. Front..

[57] Ciulla M.G., Marchini A., Gazzola J., Sambrotta M., Gelain F. (2023). Low-Power Microwaves: A Cell-Compatible Physical Treatment to Enhance Self-Assembling Peptides Mechanical Propertie. Nanoscale.

[58] Ma P.X. (2008). Biomimetic materials for tissue engineering. Adv. Drug Deliv. Rev..

[59] Ma P.X. (2004). Scaffolds for tissue fabrication. Mater. Today.

[60] Chan B.P., Leong K.W. (2008). Scaffolding in tissue engineering: General approaches and tissue-specific considerations. Eur. Spine J..

[61] Montaseri Z., Abolmaali S.S., Tamaddon A.M., Farvadi F. (2023). Composite silk fibroin hydrogel scaffolds for cartilage tissue regeneration. J. Drug Deliv. Sci. Technol..

[62] Wess T.J. (2005). Collagen Fibril Form and Function. Adv. Protein Chem..

[63] Hodge A.J., Petruska J.A., Ramachandran G.N. (1963). Recent studies with the electron microscope on ordered aggregates of the tropocollagen molecules. Aspects of Protein Structure.

[64] Ottani V., Raspanti M., Ruggeri A. (2001). Collagen structure and functional implications. Micron.

[65] Abune L., Lee K., Wang Y. (2022). Development of a Biomimetic Extracellular Matrix with Functions of Protein Sequestration and Cell Attachment Using Dual Aptamer-Functionalized Hydrogels. ACS Biomater. Sci. Eng..

[66] Hoare T.R., Kohane D.S. (2008). Hydrogels in drug delivery: Progress and challenges. Polymer.

[67] Richter A., Paschew G., Klatt S., Lienig J., Arndt K., Adler H.P. (2008). Review on Hydrogel-based pH Sensors and Microsensors. Sensors.

[68] Tan H., Marra K.G. (2010). Injectable, Biodegradable Hydrogels for Tissue Engineering Applications. Materials.

[69] Nicolson P.C. (2001). Soft contact lens polymers: An evolution. Biomacromolecules.

[70] Films G.H., Xu Y., Lin Z., Huang X., Liu Y., Huang Y., Duan X. (2013). Flexible Solid-State Supercapacitors Based on Three-Dimensional. ACS Nano.

[71] Caló E., Khutoryanskiy V.V. (2015). Biomedical applications of hydrogels: A review of patents and commercial products. Eur. Polym. J..

[72] Burdick J.A., Prestwich G.D. (2011). Hyaluronic acid hydrogels for biomedical applications. Adv. Mater..

[73] Hoffman A.S. (2012). Hydrogels for biomedical applications. Adv. Drug Deliv. Rev..

[74] Suresh Kumar N., Padma Suvarna R., Chandra Babu Naidu K., Banerjee P., Ratnamala A., Manjunatha H. (2020). A review on biological and biomimetic materials and their applications. Appl. Phys. A Mater. Sci. Process..

[75] Cecen B., Bal-Ozturk A., Yasayan G., Alarcin E., Kocak P., Tutar R., Kozaci L.D., Shin S.R., Miri A.K. (2022). Selection of natural biomaterials for micro-tissue and organ-on-chip models. J. Biomed. Mater. Res. Part A.

[76] Li Y., Tian H., Chen X. (2015). Hyaluronic acid based injectable hydrogels for localized and sustained gene delivery. J. Control. Release.

[77] Muşat V., Anghel E.M., Zaharia A., Atkinson I., Mocioiu O.C., Buşilă M., Alexandru P. (2021). A chitosan–agarose polysaccharide-based hydrogel for biomimetic remineralization of dental enamel. Biomolecules.

[78] Tran T.T., Hamid Z.A., Cheong K.Y. (2018). A Review of Mechanical Properties of Scaffold in Tissue Engineering: Aloe Vera Composites. Journal of Physics: Conference Series.

[79] Khan Y., Bashir S., Hina M., Ramesh S., Ramesh K., Lahiri I. (2020). Effect of Salt Concentration on Poly (Acrylic Acid) Hydrogel Electrolytes and their Applications in Supercapacitor. J. Electrochem. Soc..

[80] Wang H., Chen Z., Cheng S., Li R., Pan X., Zhang C., Gu H., Xie A., Dong W. (2022). Synthesis of cationic hydrogels with tunable physicochemical properties for antibacterial applications. Eur. Polym. J..

[81] Wang T., Jones J.D., Niyonshuti I.I., Agrawal S., Gundampati R.K., Kumar T.K.S., Quinn K.P., Chen J. (2019). Biocompatible, Injectable Anionic Hydrogels Based on Poly(Oligo Ethylene Glycol Monoacrylate- co -Acrylic Acid) for Protein Delivery. Adv. Ther..

[82] Chang R.Y.K., Okamoto Y., Morales S., Kutter E., Chan H.-K. (2021). Hydrogel formulations containing non-ionic polymers for topical delivery of bacteriophages. Int. J. Pharm..

[83] He H., Xiao Z., Zhou Y., Chen A., Xuan X., Li Y., Guo X., Zheng J., Xiao J., Wu J. (2019). Zwitterionic poly(sulfobetaine methacrylate) hydrogels with optimal mechanical properties for improving wound healing in vivo. J. Mater. Chem. B.

[84] Iizawa T., Ninomiya T., Gotoh T., Sakohara S. (2004). Synthesis of Porous Poly(N-isopropylacrylamide) Gel Beads by Sedimentation Polymerization. Polym. J..

[85] Singh M.R., Patel S., Singh D. (2016). Natural polymer-based hydrogels as scaffolds for tissue engineering. Nanobiomaterials in Soft Tissue Engineering.

[86] Krstic M., Rogic Miladinovic Z., Barudzija T., Mladenovic A., Suljovrujic E. (2022). Stimuli-responsive copolymeric hydrogels based on oligo(ethylene glycol) dimethacrylate for biomedical applications: An optimisation study of pH and thermoresponsive behaviour. React. Funct. Polym..

[87] Yan C., Altunbas A., Yucel T., Nagarkar R.P., Schneider J.P., Pochan D.J. (2010). Injectable solid hydrogel: Mechanism of shear-thinning and immediate recovery of injectable β-hairpin peptide hydrogels. Soft Matter.

[88] Ghorbanizamani F., Moulahoum H., Guler Celik E., Timur S. (2022). Ionic liquids enhancement of hydrogels and impact on biosensing applications. J. Mol. Liq..

[89] Baus R.A., Zahir-Jouzdani F., Dünnhaupt S., Atyabi F., Bernkop-Schnürch A. (2019). Mucoadhesive hydrogels for buccal drug delivery: In vitro-in vivo correlation study. Eur. J. Pharm. Biopharm..

[90] Janes K.A., Fresneau M.P., Marazuela A., Fabra A., Alonso M.J. (2001). Chitosan nanoparticles as delivery systems for doxorubicin. J. Control. Release.

[91] Ono K., Saito Y., Yura H., Ishikawa K., Kurita A., Akaike T., Ishihara M. (2000). Photocrosslinkable chitosan as a biological adhesive. J. Biomed. Mater. Res..

[92] ITO T., YEO Y., HIGHLEY C., BELLAS E., KOHANE D. (2007). Dextran-based in situ cross-linked injectable hydrogels to prevent peritoneal adhesions. Biomaterials.

[93] Riacci L., Sorriento A., Ricotti L. (2021). Genipin-based crosslinking of jellyfish collagen 3D hydrogels. Gels.

[94] Wang S., Yu L., Wang S., Zhang L., Chen L., Xu X., Song Z., Liu H., Chen C. (2022). Strong, tough, ionic conductive, and freezing-tolerant all-natural hydrogel enabled by cellulose-bentonite coordination interactions. Nat. Commun..

[95] Nasution H., Harahap H., Dalimunthe N.F., Ginting M.H.S., Jaafar M., Tan O.O.H., Aruan H.K., Herfananda A.L. (2022). Hydrogel and Effects of Crosslinking Agent on Cellulose-Based Hydrogels: A Review. Gels.

[96] Huang C., Ye Q., Dong J., Li L., Wang M., Zhang Y., Zhang Y., Wang X., Wang P., Jiang Q. (2023). Biofabrication of natural Au/bacterial cellulose hydrogel for bone tissue regeneration via in-situ fermentation. Smart Mater. Med..

[97] Massironi A., Franco A.R., Babo P.S., Puppi D., Chiellini F., Reis R.L., Gomes M.E. (2022). Development and Characterization of Highly Stable Silver NanoParticles as Novel Potential Antimicrobial Agents for Wound Healing Hydrogels. Int. J. Mol. Sci..

[98] Trappmann B., Chen C.S. (2013). How cells sense extracellular matrix stiffness: A material’s perspective. Curr. Opin. Biotechnol..

[99] Leng Y., Abdullah A., Wendt M.K., Calve S. (2019). Hyaluronic acid, CD44 and RHAMM regulate myoblast behavior during embryogenesis. Matrix Biol..

[100] Nakod P.S., Kim Y., Rao S.S. (2020). Three-dimensional biomimetic hyaluronic acid hydrogels to investigate glioblastoma stem cell behaviors. Biotechnol. Bioeng..

[101] Antich C., de Vicente J., Jiménez G., Chocarro C., Carrillo E., Montañez E., Gálvez-Martín P., Marchal J.A. (2020). Bio-inspired hydrogel composed of hyaluronic acid and alginate as a potential bioink for 3D bioprinting of articular cartilage engineering constructs. Acta Biomater..

[102] Almeida L.D.F., Babo P.S., Silva C.R., Rodrigues M.T., Hebling J., Reis R.L., Gomes M.E. (2018). Hyaluronic acid hydrogels incorporating platelet lysate enhance human pulp cell proliferation and differentiation. J. Mater. Sci. Mater. Med..

[103] Levin A., Hakala T.A., Schnaider L., Bernardes G.J.L., Gazit E., Knowles T.P.J. (2020). Biomimetic peptide self-assembly for functional materials. Nat. Rev. Chem..

[104] Issaka E., Wariboko M.A., Agyekum E.A. (2023). Synergy and Coordination between Biomimetic Nanoparticles and Biological Cells/Tissues/Organs/Systems: Applications in Nanomedicine and Prospect.

[105] Hosoyama K., Lazurko C., Muñoz M., McTiernan C.D., Alarcon E.I. (2019). Peptide-based functional biomaterials for soft-tissue repair. Front. Bioeng. Biotechnol..

[106] Pugliese R., Gelain F. (2017). Peptidic Biomaterials: From Self-Assembling to Regenerative Medicine. Trends Biotechnol..

[107] Aulisa L., Dong H., Hartgerink J.D. (2009). Self-Assembly of Multidomain Peptides: Sequence Variation Allows Control over Cross-Linking and Viscoelasticity. Biomacromolecules.

[108] Zhang S. (2020). Self-assembling peptides: From a discovery in a yeast protein to diverse uses and beyond. Protein Sci..

[109] Zhang S., Altman M. (1999). Peptide self-assembly in functional polymer science and engineering. React. Funct. Polym..

[110] Gelain F., Luo Z., Zhang S. (2020). Self-Assembling Peptide EAK16 and RADA16 Nanofiber Scaffold Hydrogel. Chem. Rev..

[111] Kisiday J., Jin M., Kurz B., Hung H., Semino C., Zhang S., Grodzinsky A.J. (2002). Self-assembling peptide hydrogel fosters chondrocyte extracellular matrix production and cell division: Implications for cartilage tissue repair. Proc. Natl. Acad. Sci. USA.

[112] Gelain F., Silva D., Caprini A., Taraballi F., Natalello A., Villa O., Nam K.T., Zuckermann R.N., Doglia S.M., Vescovi A. (2011). BMHP1-derived self-assembling peptides: Hierarchically assembled structures with self-healing propensity and potential for tissue engineering applications. ACS Nano.

[113] Ciulla M.G., Fontana F., Lorenzi R., Marchini A., Campone L., Sadeghi E., Paleari A., Sattin S., Gelain F. (2023). Novel self-assembling cyclic peptides with reversible supramolecular nanostructures. Mater. Chem. Front..

[114] Yang M., Xing R., Shen G., Yuan C., Yan X. (2019). A versatile cyclic dipeptide hydrogelator: Self-assembly and rheology in various physiological conditions. Colloids Surfaces A Physicochem. Eng. Asp..

[115] Insua I., Montenegro J. (2020). 1D to 2D Self Assembly of Cyclic Peptides. J. Am. Chem. Soc..

[116] Dehsorkhi A., Castelletto V., Hamley I.W. (2014). Self-assembling amphiphilic peptides. J. Pept. Sci..

[117] Hendricks M.P., Sato K., Palmer L.C., Stupp S.I. (2017). Supramolecular Assembly of Peptide Amphiphiles. Acc. Chem. Res..

[118] Lopez-Silva T.L., Leach D.G., Li I.-C., Wang X., Hartgerink J.D. (2019). Self-Assembling Multidomain Peptides: Design and Characterization of Neutral Peptide-Based Materials with pH and Ionic Strength Independent Self-Assembly. ACS Biomater. Sci. Eng..

[119] Marchini A., Raspa A., Pugliese R., Abd El Malek M., Pastori V., Lecchi M., Vescovi A.L., Gelain F. (2019). Multifunctionalized hydrogels foster hNSC maturation in 3D cultures and neural regeneration in spinal cord injuries. Proc. Natl. Acad. Sci. USA.

[120] Marchini A., Ciulla M.G., Antonioli B., Agnoli A., Bovio U., Visnoviz V., Bertuzzi F., Gelain F. (2023). Long-term cultures of human pancreatic islets in self-assembling peptides hydrogels. Front. Bioeng. Biotechnol..

[121] Forouharshad M., Raspa A., Marchini A., Ciulla M.G., Magnoni A., Gelain F. (2023). Biomimetic Electrospun Self-Assembling Peptide Scaffolds for Neural Stem Cell Transplantation in Neural Tissue Engineering. Pharmaceutics.

[122] Standley S.M., Toft D.J., Cheng H., Soukasene S., Chen J., Raja S.M., Band V., Band H., Cryns V.L., Stupp S.I. (2010). Induction of Cancer Cell Death by Self-assembling Nanostructures Incorporating a Cytotoxic Peptide. Cancer Res..

[123] Kumar V.B., Ozguney B., Vlachou A., Chen Y., Gazit E., Tamamis P. (2023). Peptide Self-Assembled Nanocarriers for Cancer Drug Delivery. J. Phys. Chem. B.

[124] Hernandez A., Hartgerink J.D., Young S. (2023). Self-assembling peptides as immunomodulatory biomaterials. Front. Bioeng. Biotechnol..

[125] Ciulla M.G., Gelain F. (2023). Structure-Activity Relationships of Antibacterial Peptides. Microb. Biotechnol..

[126] Yang J., An H.-W., Wang H. (2021). Self-Assembled Peptide Drug Delivery Systems. ACS Appl. Bio Mater..

[127] Tikhonova T.N., Cohen-Gerassi D., Arnon Z.A., Efremov Y., Timashev P., Adler-Abramovich L., Shirshin E.A. (2022). Tunable Self-Assembled Peptide Hydrogel Sensor for Pharma Cold Supply Chain. ACS Appl. Mater. Interfaces.

[128] Deng D., Chang Y., Liu W., Ren M., Xia N., Hao Y. (2023). Advancements in Biosensors Based on the Assembles of Small Organic Molecules and Peptides. Biosensors.

[129] Farsheed A.C., Thomas A.J., Pogostin B.H., Hartgerink J.D. (2023). 3D Printing of Self-Assembling Nanofibrous Multidomain Peptide Hydrogels. Adv. Mater..

[130] Wang X.-J., Cheng J., Zhang L.-Y., Zhang J.-G. (2022). Self-assembling peptides-based nano-cargos for targeted chemotherapy and immunotherapy of tumors: Recent developments, challenges, and future perspectives. Drug Deliv..

[131] Peng F., Zhang W., Qiu F. (2020). Self-assembling Peptides in Current Nanomedicine: Versatile Nanomaterials for Drug Delivery. Curr. Med. Chem..

[132] Das A.K., Gavel P.K. (2020). Low molecular weight self-assembling peptide-based materials for cell culture, antimicrobial, anti-inflammatory, wound healing, anticancer, drug delivery, bioimaging and 3D bioprinting applications. Soft Matter.

[133] Bong D.T., Clark T.D., Granja J.R., Ghadiri M.R. (2001). Self-Assembling Organic Nanotubes. Angew. Chemie Int. Ed..

[134] Ma M., Stoyanova M., Rademacher G., Dutcher S.K., Brown A., Zhang R. (2019). Structure of the Decorated Ciliary Doublet Microtubule. Cell.

[135] Li H., DeRosier D.J., Nicholson W.V., Nogales E., Downing K.H. (2002). Microtubule Structure at 8 Å Resolution. Structure.

[136] He Y.-X., Zhang N.-N., Li W.-F., Jia N., Chen B.-Y., Zhou K., Zhang J., Chen Y., Zhou C.-Z. (2012). N-Terminal Domain of Bombyx mori Fibroin Mediates the Assembly of Silk in Response to pH Decrease. J. Mol. Biol..

[137] Rubenstein M., Cornejo A., Nagpal R. (2014). Programmable self-assembly in a thousand-robot swarm. Science.

[138] Goldstein S.C., Campbell J.D., Mowry T.C. (2005). Programmable matter. Computer.

[139] Tolley M.T., Kalontarov M., Neubert J., Erickson D., Lipson H. (2010). Stochastic Modular Robotic Systems: A Study of Fluidic Assembly Strategies. IEEE Trans. Robot..

[140] Salleh F., Amid A., Nordin N.F.H. (2022). In Vitro Study on Collagen Application in Wound Healing: A Systematic Review. IIUM Med. J. Malaysia.

[141] Wang Y., Wang Z., Dong Y. (2023). Collagen-Based Biomaterials for Tissue Engineering. ACS Biomater. Sci. Eng..

[142] Speakman J.B., Ling S. (2021). Fibrous Proteins. Design, Synthesis, and Assembly.

[143] Chattopadhyay S., Raines R.T. (2014). Review collagen-based biomaterials for wound healing. Biopolymers.

[144] Ghodbane S.A., Dunn M.G. (2016). Physical and mechanical properties of cross-linked type I collagen scaffolds derived from bovine, porcine, and ovine tendons. J. Biomed. Mater. Res. Part A.

[145] Nayak V.V., Tovar N., Khan D., Pereira A.C., Mijares D.Q., Weck M., Durand A., Smay J.E., Torroni A., Coelho P.G. (2023). 3D Printing Type 1 Bovine Collagen Scaffolds for Tissue and In Vitro Evaluation. Gels.

[146] Davison-Kotler E., Marshall W.S., García-Gareta E. (2019). Sources of collagen for biomaterials in skin wound healing. Bioengineering.

[147] Silva T.H., Moreira-Silva J., Marques A.L.P., Domingues A., Bayon Y., Reis R.L. (2014). Marine origin collagens and its potential applications. Mar. Drugs.

[148] Rigogliuso S., Campora S., Notarbartolo M., Ghersi G. (2023). Recovery of Bioactive Compounds from Marine Organisms: Focus on the Future Perspectives for Pharmacological, Biomedical and Regenerative Medicine Applications of Marine Collagen. Molecules.

[149] Diamantides N., Wang L., Pruiksma T., Siemiatkoski J., Dugopolski C., Shortkroff S., Kennedy S., Bonassar L.J. (2017). Correlating rheological properties and printability of collagen bioinks: The effects of riboflavin photocrosslinking and pH. Biofabrication.

[150] Gaudet I.D., Shreiber D.I. (2012). Characterization of methacrylated Type-I collagen as a dynamic, photoactive hydrogel. Biointerphases.

[151] Zeugolis D.I., Paul R.G., Attenburrow G. (2008). Factors influencing the properties of reconstituted collagen fibers prior to self-assembly: Animal species and collagen extraction method. J. Biomed. Mater. Res. Part A.

[152] Ferrario C., Rusconi F., Pulaj A., Macchi R., Landini P., Paroni M., Colombo G., Martinello T., Melotti L., Gomiero C. (2020). From food waste to innovative biomaterial: Sea urchin-derived collagen for applications in skin regenerative medicine. Mar. Drugs.

[153] Carolo A., Melotti L., Zivelonghi G., Sacchetto R., Akyürek E.E., Martinello T., Venerando A., Iacopetti I., Sugni M., Martinelli G. (2023). Mutable Collagenous Tissue Isolated from Echinoderms Leads to the Production of a Dermal Template That Is Biocompatible and Effective for Wound Healing in Rats. Mar. Drugs.

[154] Xu Q., Torres J.E., Hakim M., Babiak P.M., Pal P., Battistoni C.M., Nguyen M., Panitch A., Solorio L., Liu J.C. (2021). Collagen- and hyaluronic acid-based hydrogels and their biomedical applications. Mater. Sci. Eng. R Rep..

[155] Doyle M.E., Dalgarno K., Masoero E., Ferreira A.M. (2023). Advances in biomimetic collagen mineralisation and future approaches to bone tissue engineering. Biopolymers.

[156] Campodoni E., Montanari M., Artusi C., Bassi G., Furlani F., Montesi M., Panseri S., Sandri M., Tampieri A. (2021). Calcium-Based Biomineralization: A Smart Approach for the Design of Novel Multifunctional Hybrid Materials. J. Compos. Sci..

[157] Focarete M.L., Tampieri A. (2018). Core-Shell Nanostructures for Drug Delivery and Theranostics Challenges, Strategies and Prospects for Novel Carrier Systems.

[158] Rastian Z., Pütz S., Wang Y.J., Kumar S., Fleissner F., Weidner T., Parekh S.H. (2018). Type I Collagen from Jellyfish Catostylus mosaicus for Biomaterial Applications. ACS Biomater. Sci. Eng..

[159] Wichuda J., Sunthorn C., Busarakum P. (2016). Comparison of the properties of collagen extracted from dried jellyfish and dried squid. Afr. J. Biotechnol..

[160] Cheng X., Shao Z., Li C., Yu L., Raja M.A., Liu C. (2017). Isolation, Characterization and Evaluation of Collagen from Jellyfish Rhopilema esculentum Kishinouye for Use in Hemostatic Applications. PLoS ONE.

[161] Veeruraj A., Arumugam M., Ajithkumar T., Balasubramanian T. (2015). Isolation and characterization of collagen from the outer skin of squid (*Doryteuthis singhalensis*). Food Hydrocoll..

[162] Dellaquila A., Campodoni E., Tampieri A., Sandri M. (2020). Overcoming the Design Challenge in 3D Biomimetic Hybrid Scaffolds for Bone and Osteochondral Regeneration by Factorial Design. Front. Bioeng. Biotechnol..

[163] Fernandes Patrício T.M., Panseri S., Sandri M., Tampieri A., Sprio S. (2017). New bioactive bone-like microspheres with intrinsic magnetic properties obtained by bio-inspired mineralisation process. Mater. Sci. Eng. C.

[164] Yu L., Rowe D.W., Perera I.P., Zhang J., Suib S.L., Xin X., Wei M. (2020). Intrafibrillar Mineralized Collagen-Hydroxyapatite-Based Scaffolds for Bone Regeneration. ACS Appl. Mater. Interfaces.

[165] Xing F., Chi Z., Yang R., Xu D., Cui J., Huang Y., Zhou C., Liu C. (2021). Chitin-hydroxyapatite-collagen composite scaffolds for bone regeneration. Int. J. Biol. Macromol..

[166] Song Y., Wu H., Gao Y., Li J., Lin K., Liu B., Lei X., Cheng P., Zhang S., Wang Y. (2020). Zinc Silicate/Nano-Hydroxyapatite/Collagen Scaffolds Promote Angiogenesis and Bone Regeneration via the p38 MAPK Pathway in Activated Monocytes. ACS Appl. Mater. Interfaces.

[167] Richardson T.P., Murphy W.L., Mooney D.J. (2001). Polymeric delivery of proteins and plasmid DNA for tissue engineering and gene therapy. Crit. Rev. Eukaryot. Gene Expr..

[168] A Biomimetic and Bioactive Scaffold with Intelligently Pulsatile Teriparatide Delivery for Local and Systemic Osteoporosis Regeneration—ScienceDirect. https://www.sciencedirect.com/science/article/pii/S2452199X22001402.

[169] Zielińska A., Karczewski J., Eder P., Kolanowski T., Szalata M., Wielgus K., Szalata M., Kim D., Shin S.R., Słomski R. (2023). Scaffolds for drug delivery and tissue engineering: The role of genetics. J. Control. Release.

[170] Flemming R.G., Murphy C.J., Abrams G.A., Goodman S.L., Nealey P.F. (1999). Effects of synthetic micro- and nano-structured surfaces on cell behavior. Biomaterials.

[171] Matsuzaka K., Walboomers X.F., De Ruijter J.E., Jansen J.A. (1999). The effect of poly-L-lactic acid with parallel surface micro on groove on osteoblast-like cells in vitro. Biomaterials.

[172] Di Pompo G., Liguori A., Carlini M., Avnet S., Boi M., Baldini N., Focarete M.L., Bianchi M., Gualandi C., Graziani G. (2023). Electrospun fibers coated with nanostructured biomimetic hydroxyapatite: A new platform for regeneration at the bone interfaces. Biomater. Adv..

[173] Mohammadalipour M., Asadolahi M., Mohammadalipour Z., Behzad T., Karbasi S. (2023). Plasma surface modification of electrospun polyhydroxybutyrate (PHB) nanofibers to investigate their performance in bone tissue engineering. Int. J. Biol. Macromol..

[174] Akbari N., Khorshidi S., Karkhaneh A. (2022). Effect of piezoelectricity of nanocomposite electrospun scaffold on cell behavior in bone tissue engineering. Iran. Polym. J..

[175] Chakraborty R., Anoop A.G., Thakur A., Mohanta G.C., Kumar P. (2023). Strategies To Modify the Surface and Bulk Properties of 3D-Printed Solid Scaffolds for Tissue Engineering Applications. ACS Omega.

[176] Shopova D., Yaneva A., Bakova D., Mihaylova A., Kasnakova P., Hristozova M., Sbirkov Y., Sarafian V., Semerdzhieva M. (2023). (Bio)printing in Personalized Medicine—Opportunities and Potential Benefits. Bioengineering.

[177] Shi Y., Deng T., Peng Y., Qin Z., Ramalingam M., Pan Y., Chen C., Zhao F., Cheng L., Liu J. (2023). Effect of Surface Modification of PEEK Artificial Phalanx by 3D Printing on its Biological Activity. Coatings.

[178] Chandra S. (2020). Natural and Synthetic Polymers. Polymers in Concrete.

[179] Grigora M.-E., Terzopoulou Z., Baciu D., Steriotis T., Charalambopoulou G., Gounari E., Bikiaris D.N., Tzetzis D. (2023). 3D printed poly(lactic acid)-based nanocomposite scaffolds with bioactive coatings for tissue engineering applications. J. Mater. Sci..

[180] Mou X., Shah J., Bhattacharya R., Kalejaiye T.D., Sun B., Hsu P.-C., Musah S. (2022). A Biomimetic Electrospun Membrane Supports the Differentiation and Maturation of Kidney Epithelium from Human Stem Cells. Bioengineering.

[181] Yao T., Chen H., Wang R., Rivero R., Wang F., Kessels L., Agten S.M., Hackeng T.M., Wolfs T.G.A.M., Fan D. (2023). Thiol-ene conjugation of a VEGF peptide to electrospun scaffolds for potential applications in angiogenesis. Bioact. Mater..

[182] Teimouri R., Abnous K., Taghdisi S.M., Ramezani M., Alibolandi M. (2023). Surface modifications of scaffolds for bone regeneration. J. Mater. Res. Technol..

[183] Laput O.A., Vasenina I.V., Korzhova A.G., Bryuzgina A.A., Khomutova U.V., Tuyakova S.G., Akhmadeev Y.H., Shugurov V.V., Bolbasov E.N., Tverdokhlebov S.I. (2023). Effect of Nitrogen Arc Discharge Plasma Treatment on Physicochemical Properties and Biocompatibility of PLA-Based Scaffolds. Polymers.

[184] Namhongsa M., Daranarong D., Molloy R., Ross S., Ross G.M., Tuantranont A., Boonyawan D., Tocharus J., Sivasinprasasn S., Topham P.D. (2023). Plasma surface modification of two-component composite scaffolds consisting of 3D-printed and electrospun fiber components from biodegradable PLGA and PLCL. Eur. Polym. J..

[185] Nakanishi K., Sakiyama T., Imamura K. (2001). On the adsorption of proteins on solid surfaces, a common but very complicated phenomenon. J. Biosci. Bioeng..

[186] Stivaktakis N., Nikou K., Panagi Z., Beletsi A., Leondiadis L., Avgoustakis K. (2005). Immune responses in mice of b-galactosidase adsorbed or encapsulated in poly(lactic acid) and poly(lactic-co-glycolic acid) microspheres. J. Biomed. Mater. Res. Part A.

[187] Thijssen Q., Parmentier L., Van Holsbeeck K., Ballet S., Van Vlierberghe S. (2023). Nature-Inspired Dual Purpose Strategy toward Cell-Adhesive PCL Networks: C(-linker-)RGD Incorporation via Thiol-ene Crosslinking. Biomacromolecules.

[188] Chroni A., Kafetzi M., Pispas S. (2023). Block copolymer-protein/peptide nanostructures for biomedical applications. Functional Materials in Biomedical Applications.

[189] Liu Q., Chiu A., Wang L., An D., Li W., Chen E.Y., Zhang Y., Pardo Y., McDonough S.P., Liu L. (2020). Developing mechanically robust, triazole-zwitterionic hydrogels to mitigate foreign body response (FBR) for islet encapsulation. Biomaterials.

[190] Kang M.-S., Lee G.-H., Kwon I.H., Yang M.-J., Heo M.B., Choi J.-W., Lee T.G., Yoon C.-H., Baek B., Sung M.-C. (2023). Uptake and toxicity of cerium dioxide nanoparticles with different aspect ratio. Toxicol. Lett..

[191] Fukuda S., Xu Y. (2022). A biomimetic anti-biofouling coating in nanofluidic channels. J. Mater. Chem. B.

[192] Cao Z., Gan T., Xu G., Ma C. (2019). Biomimetic Self-Renewal Polymer Brushes with Protein Resistance Inspired by Fish Skin. Langmuir.

[193] Jeong J.-O., Kim S., Park J., Lee S., Park J.-S., Lim Y.-M., Lee J.Y. (2020). Biomimetic nonbiofouling polypyrrole electrodes grafted with zwitterionic polymer using gamma rays. J. Mater. Chem. B.

[194] Liu S., Zhi J., Chen Y., Song Z., Wang L., Tang C., Li S., Lai X., Xu N., Liu T. (2022). Biomimetic modification on the microporous surface of cardiovascular materials to accelerate endothelialization and regulate intimal regeneration. Biomater. Adv..

[195] Yun X., Xiong Z., He Y., Wang X. (2020). Superhydrophobic lotus-leaf-like surface made from reduced graphene oxide through soft-lithographic duplication. RSC Adv..

[196] Wang Y., Yuan J., Chen J., Zeng Y., Yu T., Guo X., Wang S., Yang G., Li Y. (2023). A Single-Component Molecular Glass Resist Based on Tetraphenylsilane Derivatives for Electron Beam Lithography. ACS Omega.

[197] Nguyen T.D.T., Aryal S., Pitchaimani A., Park S., Key J., Aryal S. (2019). Biomimetic surface modification of discoidal polymeric particles. Nanomed. Nanotechnol. Biol. Med..

[198] Gao S., Chen S., Lu Q. (2019). Cell-imprinted biomimetic interface for intelligent recognition and efficient capture of CTCs. Biomater. Sci..

[199] Christopherson G.T., Song H., Mao H.-Q. (2009). The influence of fiber diameter of electrospun substrates on neural stem cell differentiation and proliferation. Biomaterials.

[200] Chen M., Patra P.K., Lovett M.L., Kaplan D.L., Bhowmick S. (2009). Role of electrospun fibre diameter and corresponding specific surface area (SSA) on cell attachment. J. Tissue Eng. Regen. Med..

[201] Ozbolat V., Dey M., Ayan B., Povilianskas A., Demirel M.C., Ozbolat I.T. (2018). 3D Printing of PDMS Improves Its Mechanical and Cell Adhesion Properties. ACS Biomater. Sci. Eng..

[202] Wu Z., Li Q., Xie S., Shan X., Cai Z. (2020). In vitro and in vivo biocompatibility evaluation of a 3D bioprinted gelatin-sodium alginate/rat Schwann-cell scaffold. Mater. Sci. Eng. C.

[203] Wu C.A., Zhu Y., Woo Y.J. (2023). Advances in 3D Bioprinting: Techniques, Applications, and Future Directions for Cardiac Tissue Engineering. Bioengineering.

[204] Gu Z., Fu J., Lin H., He Y. (2020). Development of 3D bioprinting: From printing methods to biomedical applications. Asian J. Pharm. Sci..

[205] Santoni S., Gugliandolo S.G., Sponchioni M., Moscatelli D., Colosimo B.M. (2022). 3D bioprinting: Current status and trends—A guide to the literature and industrial practice. Bio-Design Manuf..

[206] Nadernezhad A., Caliskan O.S., Topuz F., Afghah F., Erman B., Koc B. (2019). Nanocomposite Bioinks Based on Agarose and 2D Nanosilicates with Tunable Flow Properties and Bioactivity for 3D Bioprinting. ACS Appl. Bio Mater..

[207] Alarçin E., İzbudak B., Yüce Erarslan E., Domingo S., Tutar R., Titi K., Kocaaga B., Guner F.S., Bal-Öztürk A. (2023). Optimization of methacrylated gelatin /layered double hydroxides nanocomposite cell-laden hydrogel bioinks with high printability for 3D extrusion bioprinting. J. Biomed. Mater. Res. Part A.

[208] Amukarimi S., Mozafari M. (2021). 4D bioprinting of tissues and organs. Bioprinting.

[209] Díaz-Payno P.J., Kalogeropoulou M., Muntz I., Kingma E., Kops N., D’Este M., Koenderink G.H., Fratila-Apachitei L.E., van Osch G.J.V.M., Zadpoor A.A. (2023). Swelling-Dependent Shape-Based Transformation of a Human Mesenchymal Stromal Cells-Laden 4D Bioprinted Construct for Cartilage Tissue Engineering. Adv. Healthc. Mater..

[210] Kitana W., Apsite I., Hazur J., Boccaccini A.R., Ionov L. (2023). 4D Biofabrication of T-Shaped Vascular Bifurcation. Adv. Mater. Technol..

[211] Mea H., Wan J. (2022). Microfluidics-enabled functional 3D printing. Biomicrofluidics.

[212] Li Y.B., Sodja C., Rukhlova M., Nhan J., Poole J.J.A., Allen H., Yimer S., Baumann E., Bedford E., Prazak H. (2022). Microfluidic-Based 3D Bioprinting of Vascular Endothelial Networks Using Alginate-Collagen Based Biomaterials. SSRN Electron. J..

[213] Wang D., Maharjan S., Kuang X., Wang Z., Mille L.S., Tao M., Yu P., Cao X., Lian L., Lv L. (2022). Microfluidic bioprinting of tough hydrogel-based vascular conduits for functional blood vessels. Sci. Adv..

[214] Yin Y., Vázquez-Rosado E.J., Wu D., Viswananthan V., Farach A., Farach-Carson M.C., Harrington D.A. (2023). Microfluidic coaxial 3D bioprinting of cell-laden microfibers and microtubes for salivary gland tissue engineering. Biomater. Adv..

[215] You S., Xiang Y., Hwang H.H., Berry D.B., Kiratitanaporn W., Guan J., Yao E., Tang M., Zhong Z., Ma X. (2023). High cell density and high-resolution 3D bioprinting for fabricating vascularized tissues. Sci. Adv..

[216] Rajput M., Mondal P., Yadav P., Chatterjee K. (2022). Light-based 3D bioprinting of bone tissue scaffolds with tunable mechanical properties and architecture from photocurable silk fibroin. Int. J. Biol. Macromol..

[217] Kumari S., Mondal P., Chatterjee K. (2022). Digital light processing-based 3D bioprinting of κ-carrageenan hydrogels for engineering cell-loaded tissue scaffolds. Carbohydr. Polym..

[218] Wang J., Chen W., Xiao X., Xu Y., Li C., Jia X., Meng M.Q.-H. (2021). A survey of the development of biomimetic intelligence and robotics. Biomim. Intell. Robot..

[219] Whitley D. (1994). A genetic algorithm tutorial. Stat. Comput..

[220] Dorigo M., Di Caro G. (1999). Ant colony optimization: A new meta-heuristic. Proceedings of the 1999 Congress on Evolutionary Computation-CEC99 (Cat. No. 99TH8406).

[221] Freitas A.A. (2002). Data Mining and Knowledge Discovery with Evolutionary Algorithms.

[222] Lam B., Ciesielski V. (2004). Discovery of Human-Competitive Image Texture Feature Extraction Programs Using Genetic Programming. Genetic and Evolutionary Computation Conference.

[223] Ahnert S.E., Marsh J.A., Hernández H., Robinson C.V., Teichmann S.A. (2015). Principles of assembly reveal a periodic table of protein complexes. Science.

[224] Dasgupta D., Michalewicz Z. (2013). Evolutionary Algorithms in Engineering Applications.

[225] Badini S., Regondi S., Pugliese R. (2023). Unleashing the Power of Artificial Intelligence in Materials Design. Materials.

[226] Ball P. (2019). Using artificial intelligence to accelerate materials development. MRS Bull..

[227] Raabe D., Mianroodi J.R., Neugebauer J. (2023). Accelerating the design of compositionally complex materials via physics-informed artificial intelligence. Nat. Comput. Sci..

[228] Rao Z., Tung P.-Y., Xie R., Wei Y., Zhang H., Ferrari A., Klaver T.P.C., Körmann F., Sukumar P.T., Kwiatkowski da Silva A. (2022). Machine learning–enabled high-entropy alloy discovery. Science.

[229] Kaufmann K., Maryanovsky D., Mellor W.M., Zhu C., Rosengarten A.S., Harrington T.J., Oses C., Toher C., Curtarolo S., Vecchio K.S. (2020). Discovery of high-entropy ceramics via machine learning. npj Comput. Mater..

[230] Badini S., Regondi S., Frontoni E., Pugliese R. (2023). Assessing the capabilities of ChatGPT to improve additive manufacturing troubleshooting. Adv. Ind. Eng. Polym. Res..

[231] Agathokleous E., Saitanis C.J., Fang C., Yu Z. (2023). Use of ChatGPT: What does it mean for biology and environmental science?. Sci. Total Environ..

[232] Cheng K., Guo Q., He Y., Lu Y., Gu S., Wu H. (2023). Exploring the Potential of GPT-4 in Biomedical Engineering: The Dawn of a New Era. Ann. Biomed. Eng..

[233] Purkait M.K., Sinha M.K., Mondal P., Singh R. (2018). Biologically Responsive Membranes. Interface Science and Technology.

[234] Kim J.H., Lee S.J. (2016). A Biomimetic Strategy to Design Biomaterials for In Situ Tissue Regeneration. In Situ Tissue Regeneration.

[235] Lee J.Y., Bashur C.A., Milroy C.A., Forciniti L., Goldstein A.S., Schmidt C.E. (2012). Nerve Growth Factor-Immobilized Electrically Conducting Fibrous Scaffolds for Potential Use in Neural Engineering Applications. IEEE Trans. Nanobiosci..

[236] Rashid M., Roni M.A., Rahman M. (2021). Clinical status of bioinspired and biomimetic materials. Bioinspired and Biomimetic Materials for Drug Delivery.

[237] Liu S., Yu J.-M., Gan Y.-C., Qiu X.-Z., Gao Z.-C., Wang H., Chen S.-X., Xiong Y., Liu G.-H., Lin S.-E. (2023). Biomimetic natural biomaterials for tissue engineering and regenerative medicine: New biosynthesis methods, recent advances, and emerging applications. Mil. Med. Res..

[238] Qiu Z.-Y., Cui Y., Tao C.-S., Zhang Z.-Q., Tang P.-F., Mao K.-Y., Wang X.-M., Cui F.-Z. (2015). Mineralized Collagen: Rationale, Current Status, and Clinical Applications. Materials.

[239] Song T.-X., Hu Y.-L., He Z.-M., Cui Y., Ding Q., Qiu Z.-Y. (2019). Clinical Applications of the Mineralized Collagen. Mineralized Collagen Bone Graft Substitutes.

[240] Kantak M.N., Bharate S.S. (2022). Analysis of clinical trials on biomaterial and therapeutic applications of chitosan: A review. Carbohydr. Polym..

